# Multi‐targeting of viral RNAs with synthetic *trans*‐acting small interfering RNAs enhances plant antiviral resistance

**DOI:** 10.1111/tpj.14466

**Published:** 2019-09-16

**Authors:** Alberto Carbonell, Purificación Lisón, José‐Antonio Daròs

**Affiliations:** ^1^ Instituto de Biología Molecular y Celular de Plantas Consejo Superior de Investigaciones Científicas‐Universitat Politècnica de València 46022 Valencia Spain

**Keywords:** syn‐tasiRNA, antiviral resistance, amiRNA, RNA silencing, *Solanum lycopersicum*, *Tomato spotted wilt virus*

## Abstract

RNA interference (RNAi)‐based tools are used in multiple organisms to induce antiviral resistance through the sequence‐specific degradation of target RNAs by complementary small RNAs. In plants, highly specific antiviral RNAi‐based tools include artificial microRNAs (amiRNAs) and synthetic *trans*‐acting small interfering RNAs (syn‐tasiRNAs). syn‐tasiRNAs have emerged as a promising antiviral tool allowing for the multi‐targeting of viral RNAs through the simultaneous expression of several syn‐tasiRNAs from a single precursor. Here, we compared in tomato plants the effects of an amiRNA construct expressing a single amiRNA and a syn‐tasiRNA construct expressing four different syn‐tasiRNAs against *Tomato spotted wilt virus* (TSWV), an economically important pathogen affecting tomato crops worldwide. Most of the syn‐tasiRNA lines were resistant to TSWV, whereas the majority of the amiRNA lines were susceptible and accumulated viral progenies with mutations in the amiRNA target site. Only the two amiRNA lines with higher amiRNA accumulation were resistant, whereas resistance in syn‐tasiRNA lines was not exclusive of lines with high syn‐tasiRNA accumulation. Collectively, these results suggest that syn‐tasiRNAs induce enhanced antiviral resistance because of the combined silencing effect of each individual syn‐tasiRNA, which minimizes the possibility that the virus simultaneously mutates all different target sites to fully escape each syn‐tasiRNA.

## Significance Statement

This work represents the successful application of syn‐tasiRNAs in a crop to induce high resistance against an economically important virus. Syn‐tasiRNAs outcompete amiRNAs in antiviral resistance: the possibility of co‐expressing multiple syn‐tasiRNAs from a single precursor allows for the simultaneous multi‐targeting of viral RNAs, which limits the ability of the virus to mutate target sites.

## Introduction

RNA interference (RNAi) refers to the sequence‐specific degradation of target RNA by complementary small RNAs (sRNAs) (Fire *et al*., 1998). Gene silencing via RNAi has been a central tool in modern molecular biology, allowing specific genes to be ‘switched off’ for both basic and applied studies in multiple organisms. In humans, antiviral therapies using small interfering RNAs (siRNAs) were one of the first RNAi‐based medical applications (for a recent review, see Levanova and Poranen, 2018). Generally, antiviral siRNAs are designed to directly target complementary viral RNAs but can also be produced for blocking virus entry through the silencing of host genes. siRNA duplexes of 21–23 nucleotides (nt) are produced by chemical or enzymatic synthesis, or through the expression of short hairpin RNA (hpRNA) precursors that do not elicit the non‐specific interferon responses. Typically, one of the strands of the duplex, the guide RNA, is loaded into an RNA‐induced silencing complex (RISC), including an ARGONAUTE (AGO) protein, and directs RISC to bind and silence perfect complementary viral RNAs through AGO‐mediated endonucleolytic cleavage (Ding, 2010).

In plants, classic antiviral RNAi approaches, such as virus‐induced gene silencing (VIGS) and hairpin‐based silencing, have been extensively used and are based on the activity of longer double‐stranded RNA (dsRNA) or hpRNA precursors including sequences of the target virus. The large populations of siRNA species produced from this class of precursors can compromise the specificity of the induced silencing, however, if cellular transcripts sharing high sequence complementarity with that of certain siRNAs are accidentally targeted. The limited specificity of these approaches was overcome by the development of ‘second‐generation RNAi’ strategies based on artificial sRNAs, such as artificial miRNAs (amiRNAs) and synthetic *trans*‐acting siRNAs (syn‐tasiRNAs) (Carbonell, 2017). Artificial sRNAs are 21‐nt sRNAs designed to be highly specific, and with high sequence complementarity with target RNA(s). In particular, mismatches at positions 2–13 from the artificial sRNA 5′ end are avoided, as it is well known that mismatches in this so‐called ‘mismatch‐sensitive region’ drastically reduce the silencing activity of sRNAs (Schwab *et al*., 2005; Fahlgren and Carrington, 2010). Artificial sRNAs are produced *in planta* by expressing a functional miRNA or tasiRNA precursor with modified miRNA/miRNA* or tasiRNA sequences, respectively. amiRNA precursors are processed by DICER‐LIKE 1 (DCL1) to produce amiRNA/amiRNA* duplexes. syn‐tasiRNA precursors are first cleaved by an miRNA–AGO complex. A cleavage fragment is used by RNA‐DEPENDENT RNA POLYMERASE 6 (RDR6) to synthesize a dsRNA sequentially processed by DCL4 into syn‐tasiRNA duplexes in register with the miRNA cleavage site. For both classes of artificial sRNAs, the guide strand is selectively incorporated into an AGO protein, generally AGO1, to direct the specific cleavage of complementary target RNAs, whereas the other strand of the duplex is generally degraded. amiRNAs were first used to confer antiviral resistance in transgenic plants (Niu *et al*., 2006); however, virus sequence variants accumulating mutations in the amiRNA target site (TS) may overcome this resistance (Simon‐Mateo and Garcia, 2006; Lin *et al*., 2009; Lafforgue *et al*., 2011). More recently, syn‐tasiRNAs have emerged as a promising antiviral tool because of their unique multiplexing capability (Zhang, 2014; Carbonell, 2019b) as well as for the availability of high‐throughput cloning strategies and automated design tools for the simple generation of syn‐tasiRNA constructs (Carbonell *et al*., 2014; Carbonell, 2019a). The simultaneous co‐expression from a single precursor of several syn‐tasiRNAs capable of targeting multiple sites within a single viral RNA or within different viral RNAs should induce more effective, durable, and broad antiviral resistance (Carbonell *et al*., 2016). Indeed, syn‐tasiRNAs have been reported to confer resistance against different viral species or isolates in model plants such as *Arabidopsis thaliana* and *Nicotiana benthamiana* (Chen *et al*., 2016; Carbonell *et al*., 2019); however, the antiviral activity of syn‐tasiRNAs has neither been examined in a crop nor systematically compared with that of amiRNAs.

*Tomato spotted wilt virus* (TSWV) is one of the top plant viruses in terms of scientific and economic importance, causing high yield losses in tomato and pepper plants worldwide (Scholthof *et al*., 2011). TSWV, the type species of the genus *Tospovirus* (family *Bunyaviridae*), has an RNA genome consisting of three negative or ambisense single‐stranded RNAs, named segments L, M and S, encoding the viral RNA‐dependent RNA polymerase (RdRp), the putative movement protein NSm and the structural proteins Gn/Gc, and the nucleocapsid N protein and the silencing suppressor NSs, respectively (Kormlink, 2011). Previously, an efficient high‐throughput methodology was used in *N. benthamiana* to identify several amiRNAs with high antiviral activity against TSWV (Carbonell *et al*., 2019). A comparative analysis between the most effective amiRNA construct and a syn‐tasiRNA construct including the four most active amiRNA sequences revealed that both were similarly efficient against two different TSWV isolates when transiently expressed in leaves before virus inoculation (Carbonell *et al*., 2019).

Here, we aimed to systematically analyze and compare the anti‐TSWV resistance induced by these same amiRNA and syn‐tasiRNA sequences in the TSWV natural host, *Solanum lycopersicum* (tomato). AmiRNA and syn‐tasiRNA constructs were introduced independently into tomato plants to generate multiple stably transformed lines that were analyzed for anti‐TSWV resistance. Infectivity assays revealed that the majority of the syn‐tasiRNA lines were resistant to TSWV, and only lines with particularly low syn‐tasiRNA accumulation were infected. In contrast, most of the amiRNA lines were susceptible, and only the two lines with higher amiRNA accumulation were resistant. A systematic analysis of TS sequences in viral progenies from infected plants revealed the emergence of virus sequence variants, including TS mutations exclusively in susceptible amiRNA lines. TS mutations were all nucleotide substitutions, and generally clustered in the mismatch‐sensitive region, led to mismatches and did not alter the amino acid composition of the corresponding viral protein. Collectively, our results indicate that subinhibitory amiRNA accumulation favors the emergence of TS mutations in replicating TSWV, while simultaneous multi‐targeting of viral RNAs with several syn‐tasiRNAs limits the ability of the virus to mutate TSs, thus enhancing plant resistance.

## Results

### Generation of a construct for expressing anti‐TSWV syn‐tasiRNAs in tomato plants

Previously, an efficient methodology was developed in the model plant *N. benthamiana* for the fast‐forward identification of artificial sRNAs with high antiviral or antiviroid activity (Carbonell and Daros, 2017, 2019; Carbonell *et al*., 2019). In particular, this methodology was used to reveal the high antiviral activity of four *TAS1c*‐based anti‐TSWV syn‐tasiRNAs (syn‐tasiR‐TSWV), transiently expressed from the *35:syn‐tasiR‐TSWV* construct in *N. benthamiana* leaves that were subsequently inoculated with an infectious TSWV extract (Carbonell *et al*., 2019). Importantly: (i) the co‐expression of a construct producing miR173 (*35S:miR173*) was required for triggering syn‐tasiRNA biogenesis from *35S:syn‐tasiR‐TSWV*‐derived *TAS1c* transcripts, as miR173 is only present in *A. thaliana* and close relatives; and (ii) the selected syn‐tasiRNA sequences were designed with the p‐sams tool (Fahlgren *et al*., 2016) to be highly specific, without any predicted off‐targets in *S. lycopersicum* (Carbonell *et al*., 2019).

**Figure 1:**
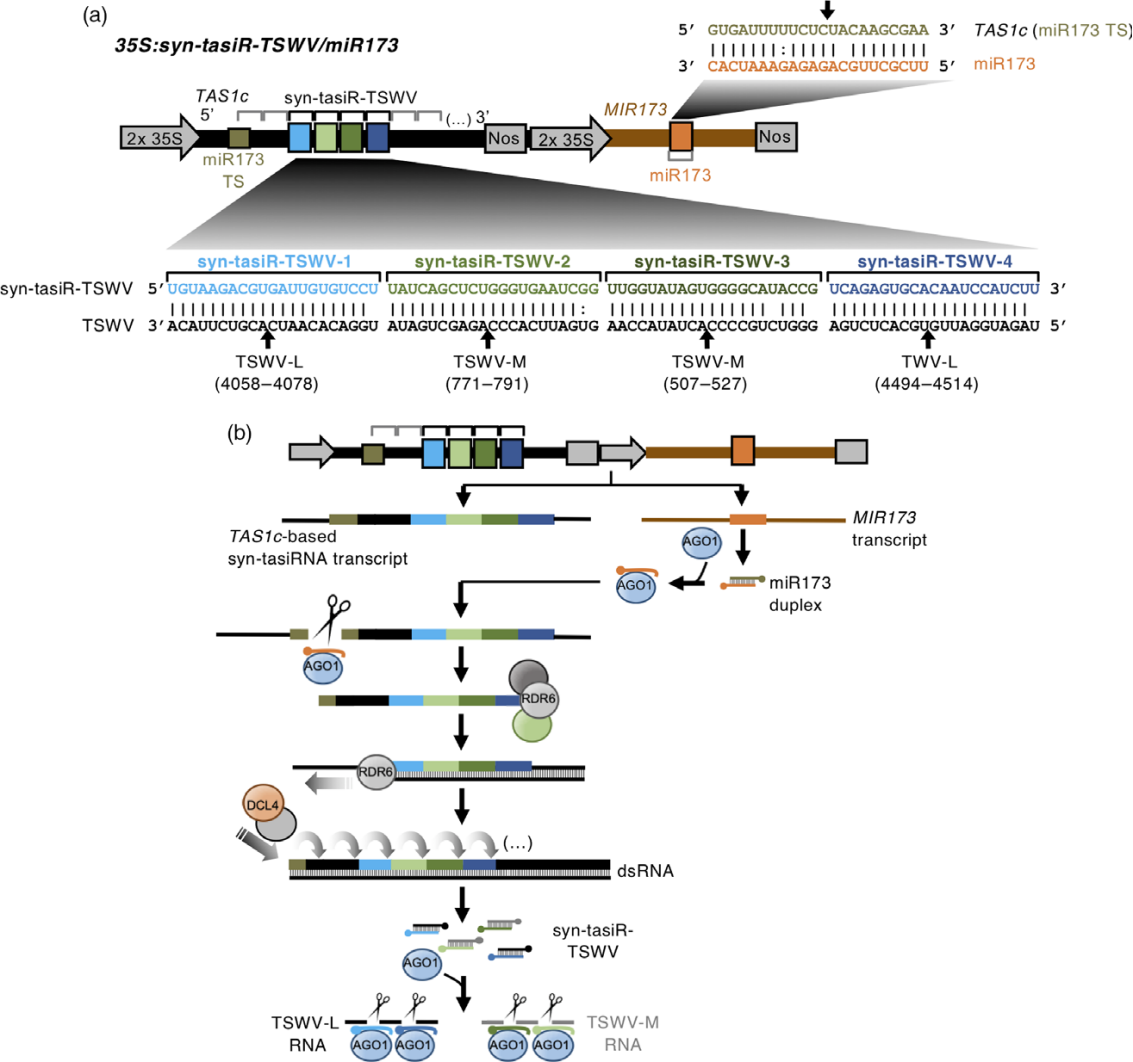
Details and functions of the anti‐*Tomato spotted wilt virus* (TSWV) synthetic *trans*‐acting small interfering RNA (syn‐tasiRNA) construct. (a) Diagram of the *35S:syn‐tasiR‐TSWV/miR173* construct. The *TAS1c* precursor from *Arabidopsis thaliana* (in black) was engineered to produce four different anti‐TSWV syn‐tasiRNAs (in light or dark blue or light or dark green boxes). tasiRNA and syn‐tasiRNA positions in *TAS1c* are indicated with grey and black brackets, respectively, and the miR173 target site (TS) is shown with a green square box. A cassette including *A. thaliana MIR173* precursor (in brown) to generate miR173 (orange box) was inserted downstream the syn‐tasiRNA cassette. Both cassettes contain a double *35S* promoter and an Nos terminator (in grey arrows or boxes, respectively) for *in planta* expression. Base pairing between syn‐tasiRNA and TSWV nucleotides is shown. Specific cleavage sites in target sites located in L or M segments in TSWV RNAs are indicated with black arrows, with TS coordinates indicated in brackets. miR173/*TAS1c* base‐pairing diagram is also shown, with the miR173‐guided cleavage site in *TAS1c* indicated with a black arrow. (b) Diagram showing the biogenesis and activities of *35S:syn‐tasiR‐TSWV/miR173* products. Details are the same as in (a).

As TSWV is an economically important pathogen reducing tomato yields worldwide, here we aimed to explore whether such syn‐tasiRNAs could be used to induce high anti‐TSWV resistance when stably expressed in tomato plants. As miR173 is not present in *S. lycopersicum*, a DNA cassette including the complete *MIR173* precursor sequence flanked by promoter and terminator sequences was added downstream of the syn‐tasiRNA cassette in the previously described 35S:syn‐tasiR‐TSWV construct (Carbonell *et al*., 2019), to generate *35S:syn‐tasiR‐TSWV/miR173* (Figure 1a, Figure S1). The syn‐tasiRNA cassette includes the sequence of the four most active artificial sRNAs selected in a previous screening (Carbonell *et al*., 2019). In particular, syn‐tasiR‐TSWV‐1 and syn‐tasiR‐TSWV‐3 target different sites of TSWV‐L RNAs, whereas syn‐tasiR‐TSWV‐2 and syn‐tasiR‐TSWV‐4 have target sites in TSWV‐M RNAs (Figure 1a). *35S:syn‐tasiR‐TSWV/miR173* expression in tomato transgenic plants was expected to produce miR173 to trigger syn‐tasiR‐TSWV biogenesis and subsequent silencing of TSWV RNAs (Figure 1b). Similarly, as a negative control, the *35S:syn‐tasiR‐GUS/miR173* construct (Figure S2) was generated by adding the same miR173 cassette to the previously described *35S:syn‐tasiR‐GUS* construct expressing four innocuous anti‐GUS syn‐tasiRNAs (Carbonell *et al*., 2019).

### The anti‐TSWV syn‐tasiRNA construct is functional in *N. benthamiana*

**Figure 2:**
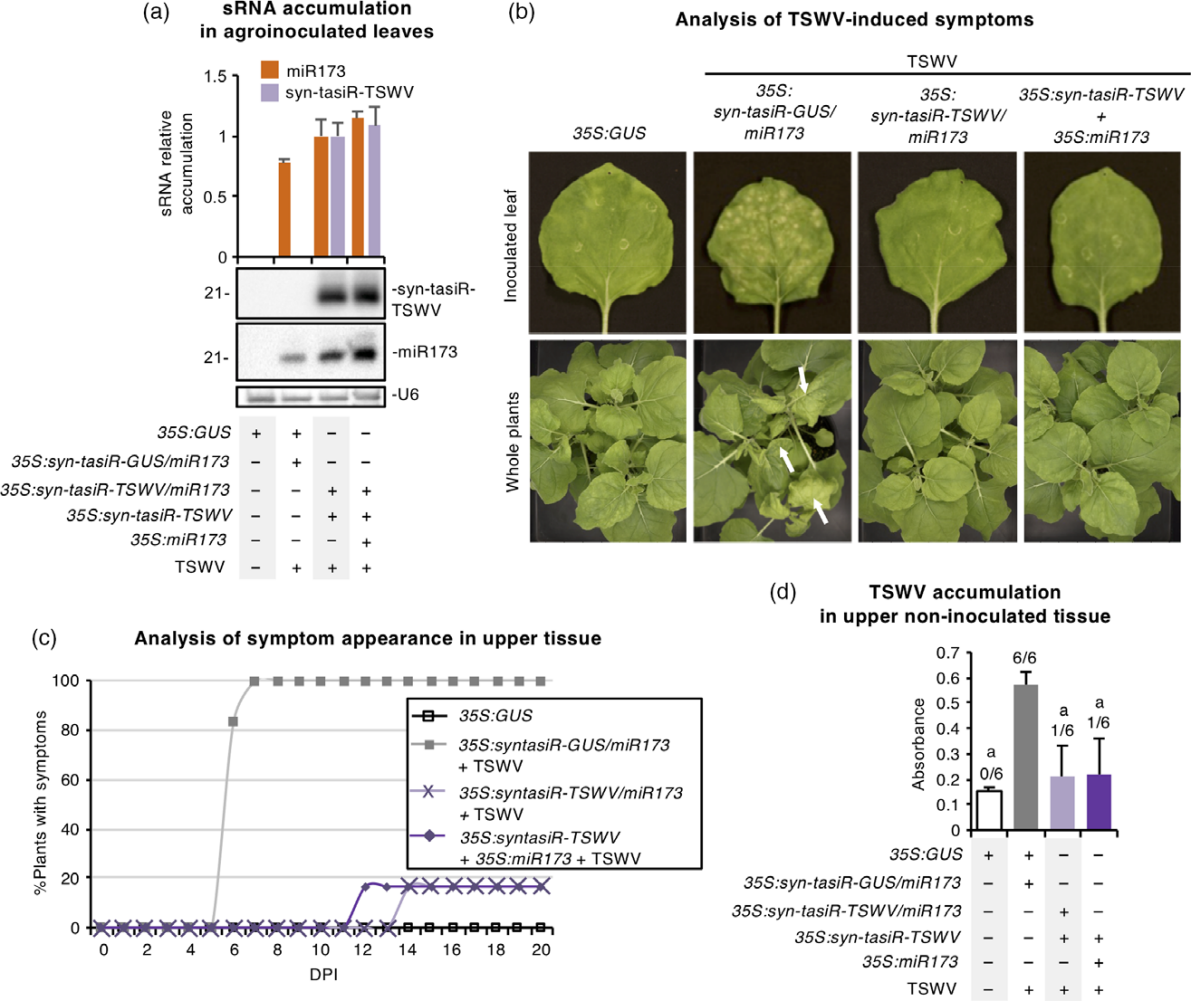
Functional analysis of the anti‐*Tomato spotted wilt virus* (TSWV) synthetic *trans*‐acting small interfering RNA (syn‐tasiRNA) construct in *Nicotiana benthamiana*. (a) Northern blot detection of syn‐tasiR‐TSWV (syn‐tasiR‐TSWV‐1 + syn‐tasiR‐TSWV‐2 + syn‐tasiR‐TSWV‐3 + syn‐tasiR‐TSWV‐4) and miR173 in RNA preparations from agroinfiltrated leaves at 2 days post‐agroinfiltration. A representative blot corresponding to one of the three biological replicates is shown. Each biological replicate is a pool of two agroinfiltrated leaves from the same plant. The U6 blot is shown as the loading control. The graph at the top shows the mean (*n *=* *3) + standard deviation (SD) syn‐tasiR‐TSWV (purple) and miR173 (orange) accumulation relative to that of lane 3 (*P *>* *0.01 for all pairwise Student's *t*‐test comparisons between lanes 3 and 4). Constructs were co‐expressed as indicated below the blot panels. (b) Plant photographs of agroinfiltrated leaves (further inoculated with TSWV) and of whole plants taken 7 and 20 days post‐inoculation (dpi), respectively. TSWV‐induced characteristic symptoms of leaf chlorosis and epinasty are indicated with white arrows. (c) Two‐dimensional line graph showing, for each of the six‐plant sets listed in the box, the percentage of symptomatic plants per day during 20 dpi. (d) Bar graph representing the mean (*n *=* *6) + SD absorbance obtained in double antibody sandwich enzyme‐linked immunosorbent assays on the indicated samples collected at 20 dpi, as an indirect estimate of TSWV accumulation. Bars with the letter ‘a’ are significantly different from that of sample *35S:syn‐tasiR‐GUS/miR173 *+* 35S:GUS* + TSWV (*P *<* *0.01 in pairwise Student's *t*‐test comparisons).

The functionality of the *35S:syn‐tasiR‐TSWV/miR173* construct was first analyzed in transient expression assays in *N. benthamiana*. To begin, the accumulation of anti‐TSWV syn‐tasiRNAs and miR173 was analyzed in leaves agroinfiltrated with *35S:syn‐tasiR‐TSWV/miR173*. For control purposes, the original *35S:syn‐tasiR‐syn‐tasiR‐TSWV* was co‐agroinfiltrated with *35S:miR173* as before (Carbonell *et al*., 2019). Northern blot analysis of RNA preparations obtained 2 days post‐agroinfiltration (dpa) revealed that both syn‐tasiR‐TSWV and miR173 accumulated to similar levels when miR173 was expressed *in* *cis* (from *35S:syn‐tasiR‐TSWV/miR173*) or *in* *trans* (from *35S:syn‐tasiR‐TSWV *+* 35S:MIR173*) (Figure 2a).

Next, the antiviral activity of *35S:syn‐tasiR‐TSWV/miR173* was analyzed using the previously described assay, in which *35S:syn‐tasiR‐TSWV* co‐expression with *35S:miR173* in *N. benthamiana* leaves that were inoculated 2 days later with TSWV resulted in high levels of resistance (Carbonell *et al*., 2019). Here, the antiviral effect of *35S:syn‐tasiR‐TSWV/miR173*,* 35S:syn‐tasiR‐GUS/miR173* and *35S:syn‐tasiR‐TSWV *+* 35S:miR173* was compared by agroinfiltrating six leaves each from independent plants, and then inoculating each leaf with TSWV 2 days later. To determine the antiviral activity of anti‐TSWV syn‐tasiRNAs, the appearance of characteristic TSWV‐induced symptoms in inoculated tissues (necrotic lesions) and in upper non‐inoculated tissues (leaf epinasty and chlorosis) was monitored. Finally, TSWV accumulation was analyzed in upper non‐inoculated leaves by ELISA at 20 days post‐inoculation (dpi).

Leaves agroinfiltrated with *35S:syn‐tasiR‐TSWV/miR173* or with *35S:syn‐tasiR‐TSWV *+* 35S:miR173* were symptomless, whereas leaves agroinfiltrated with *35S:syn‐tasiR‐GUS/miR173* showed multiple necrotic lesions (Figure 2b). Regarding the upper non‐inoculated tissues, all six plants infiltrated with *35S:syn‐tasiR‐GUS/miR173* displayed strong leaf epinasty and chlorosis (Figure 2b), already visible at 6–7 dpi (Figure 2c), and accumulated high levels of TSWV (Figure 2d; Table S2). In contrast, five of the six plants expressing *35S:syn‐tasiR‐TSWV/miR173* or *35S:syn‐tasiR‐TSWV *+* 35S:MIR173* neither showed symptoms nor accumulated TSWV (Figure 2c,d; Table S2). In both cases, only one plant showed symptoms and TSWV accumulation (Figure 2c,d; Table S2). Altogether, these results confirmed the functionality and antiviral effect of the *35S:syn‐tasiR‐TSWV/miR173* construct in *N. benthamiana*.

### Syn‐tasiRNAs induce high levels of anti‐TSWV resistance when stably expressed from a single construct in tomato transgenic plants

**Figure 3:**
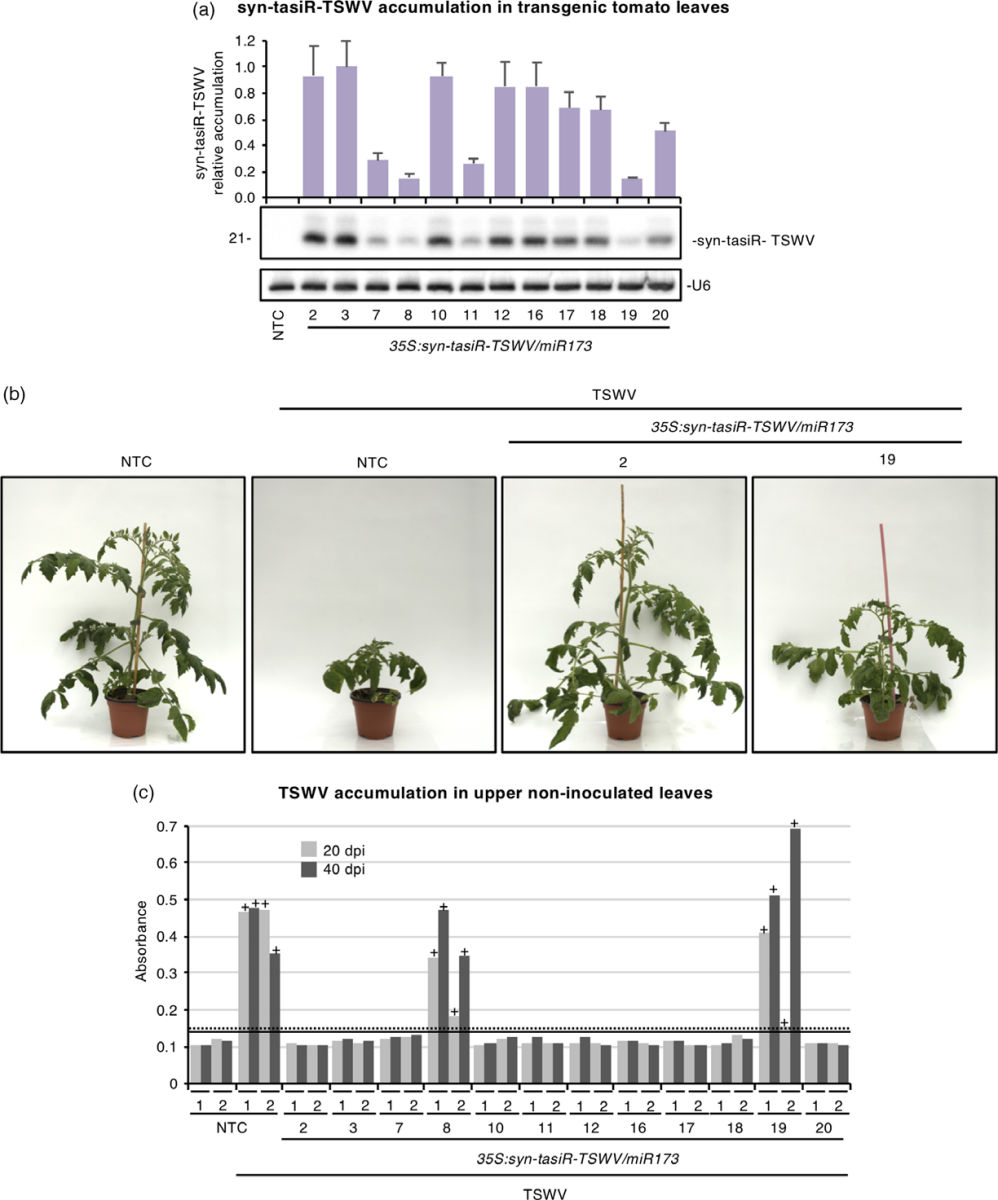
Functional analysis of the anti‐*Tomato spotted wilt virus* (TSWV) synthetic *trans*‐acting small interfering RNA (syn‐tasiRNA) construct in transgenic *Solanum lycopersicum* plants. (a) Northern blot detection of syn‐tasiR‐TSWV (syn‐tasiR‐TSWV‐1 + syn‐tasiR‐TSWV‐2 + syn‐tasiR‐TSWV‐3 + syn‐tasiR‐TSWV‐4) in RNA preparations from apical leaves of tomato transgenic lines. A representative blot corresponding to one of the three biological replicates of each line is shown. The U6 blot is shown as the loading control. The graph at the top shows the mean (*n *=* *3) + standard deviation (SD) syn‐tasiR‐TSWV accumulation relative to that of the *35S:syn‐tasiR‐TSWV/miR173‐A3* sample. (b) Photographs taken at 20 days post inoculation (dpi) of representative transgenic tomato plants expressing anti‐TSWV syn‐tasiRNAs and of non‐transgenic control (NTC) plants. (c) Bar graph representing the absorbance obtained in double antibody sandwich enzyme‐linked immunosorbent assays (DAS‐ELISAs) on indicated samples collected at 20 or 40 dpi, as an indirect estimate of TSWV accumulation. Bars with a ‘+’ sign correspond to samples that are considered DAS‐ELISA positive. Dotted and straight lines show the threshold values above which samples are considered positive for DAS‐ELISA analysis at 20 and 40 dpi, respectively.

Next, to analyse the antiviral activity of syn‐tasiRNAs in a TSWV natural host, *35S:syn‐tasiR‐TSWV/miR173* was introduced and stably expressed in *S. lycopersicum*. Twelve independent transgenic lines were generated, all of them with a phenotype that was indistinguishable from that of wild‐type tomato plants, and syn‐tasiRNA accumulation in each line was analyzed. Northern blot analysis of RNA preparations obtained from apical leaves revealed that syn‐tasiR‐TSWV accumulation was highly variable among the different lines (Figure 3a).

Two individuals of each transgenic line were inoculated with TSWV. In parallel, two non‐transgenic control (NTC) plants regenerated *in vitro* were mock‐ or TSWV‐inoculated. Both the appearance and progression of viral symptoms and TSWV accumulation were assessed at two different timepoints (20 and 40 dpi) (Table 1). Interestingly, the majority of transgenic lines (10 of 12) neither showed viral symptoms nor accumulated TSWV (Figure 3b,c; Table 1). Only two lines, *35S:syn‐tasiR‐TSWV/miR173‐8* and *35S:syn‐tasiR‐TSWV/MIR173‐19*, showed symptoms such as leaf curling, chlorosis and moderate stunting as early as 20 dpi (Figure 3b), and accumulated TSWV to high levels (Figure 3c). Importantly, these two susceptible lines were indeed the two lines with lowest syn‐tasiR‐TSWV accumulation (Figure 3a), thus indicating a dosage effect of syn‐tasiR‐TSWV accumulation. Finally, genomic TSWV RNA accumulation was analyzed at 40 dpi by reverse transcription followed by quantitative polymerase chain reaction (RT‐qPCR) in representative syn‐tasiRNA lines and NTCs (Figure S3). In all cases, RT‐qPCR results confirmed those obtained by enzyme‐linked immunosorbent assay (ELISA) analysis. Interestingly, both susceptible plants corresponding to line *35S:syn‐tasiR‐TSWV/miR173‐8* did not accumulate abnormally high levels of TSWV RNA. The comparison between TSWV RNA and protein levels in these plants suggests that syn‐tasiR‐TSWV may be acting on TSWV RNAs through endonucleolytic cleavage rather than by inducing translational repression.

**Table 1:** Symptom and virus accumulation analyses in *Solanum lycopersicum* (tomato) transgenic plants expressing antiviral syn‐tasiRNAs infected with *Tomato spotted wilt virus* (TSWV)

Tomato transgenic line	Inoculated with TSWV	Analysis at 20 dpi		Analysis at 40 dpi	
		Symptomatic plants/total	DAS‐ELISA positive/total	Symptomatic plants/total	DAS‐ELISA positive/total
NTC	−	0/2	0/2	0/2	0/2
	+	2/2	2/2	2/2	2/2
*35S:syn‐tasiR‐TSWV/miR173*					
2	+	0/2	0/2	0/2	0/2
3	+	0/2	0/2	0/2	0/2
7	+	0/2	0/2	0/2	0/2
8	+	2/2	2/2	2/2	2/2
10	+	0/2	0/2	0/2	0/2
11	+	0/2	0/2	0/2	0/2
12	+	0/2	0/2	0/2	0/2
16	+	0/2	0/2	0/2	0/2
17	+	0/2	0/2	0/2	0/2
18	+	0/2	0/2	0/2	0/2
19	+	2/2	2/2	2/2	2/2
20	+	0/2	0/2	0/2	0/2

DAS‐ELISA, double antibody sandwich enzyme‐linked immunosorbent assay; dpi, days post‐inoculation.

### A high proportion of tomato transgenic lines expressing a single anti‐TSWV amiRNA are susceptible

**Figure 4:**
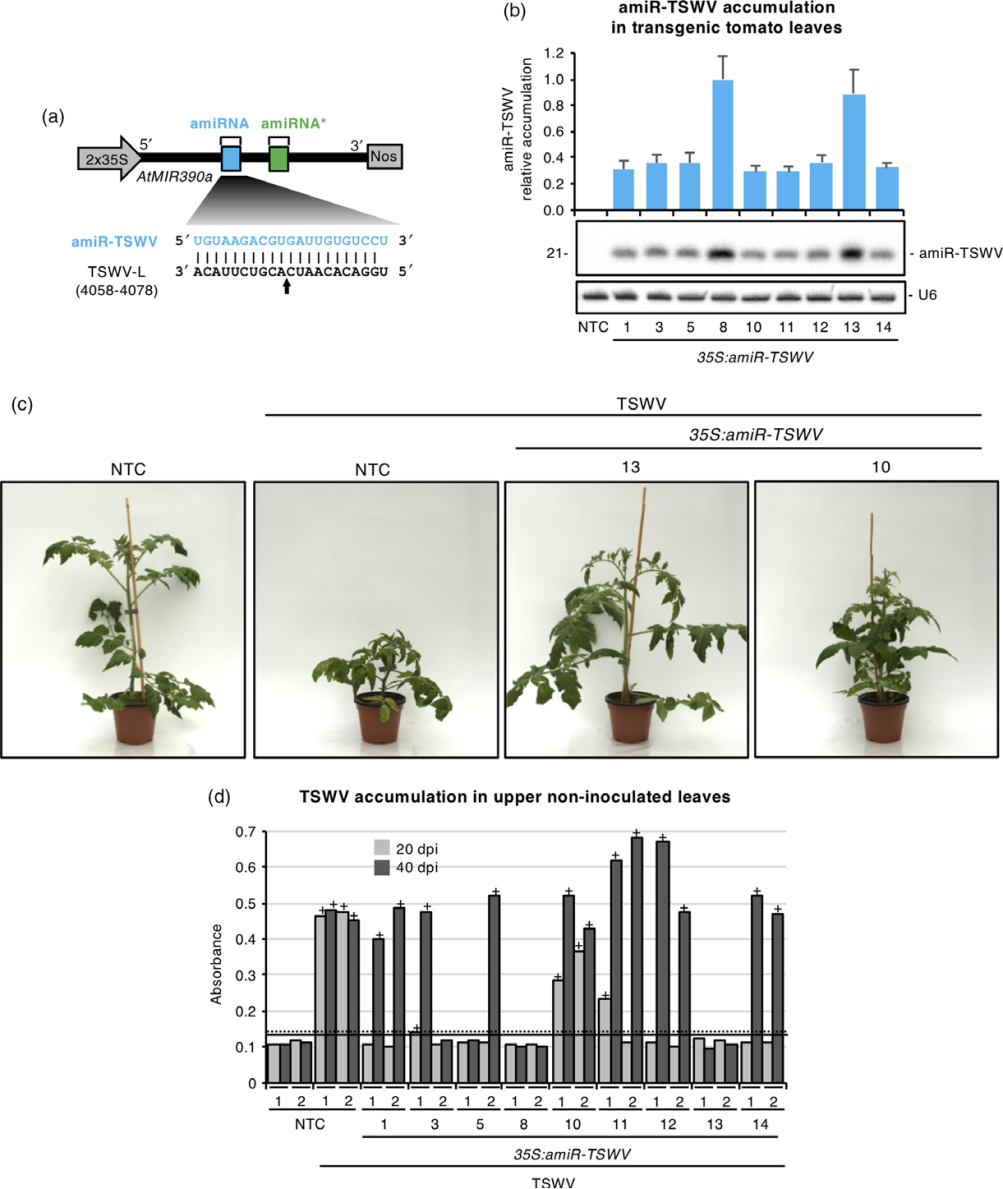
Functional analysis of the anti‐*Tomato spotted wilt virus* (TSWV) synthetic *trans*‐acting small interfering RNA (syn‐tasiRNA) construct in transgenic *Solanum lycopersicum* plants. (a) Diagram of the anti‐TSWV amiRNA construct, *35S:amiR‐TSWV*. *MIR390a* precursor from *Arabidopsis thaliana* (in black) was engineered to produce a single anti‐TSWV amiRNA. amiRNA and start strand positions in *MIR390a* are indicated with blue and green boxes, respectively. The base pairing between amiR‐TSWV and TSWV nucleotides is shown. The cleavage site located in segment L in TSWV RNAs is indicated with a black arrow, with target site coordinates indicated in brackets. Other details are the same as in Figure 1. (b) Northern blot detection of amiR‐TSWV in RNA preparations from apical leaves of tomato plants. The graph at the top shows the mean (*n *=* *3) + standard deviation (SD) amiR‐TSWV accumulation relative to that of the *35S:amiR‐TSWV‐A8* sample. Other details are the same as in Figure 3(a). (c) Photographs taken at 20 days post inoculation (dpi) of representative tomato transgenic plants expressing anti‐TSWV amiRNA and of non‐transgenic control (NTC) plants. (d) Bar graph representing the absorbance obtained in double antibody sandwich enzyme‐linked immunosorbent assays on indicated samples collected at 20 or 40 dpi, as an indirect estimate of TSWV accumulation. Other details are the same as in Figure 3(c).

To test whether syn‐tasiRNA‐mediated multi‐targeting of viral RNAs offers an advantage towards the most common single‐site targeting by amiRNAs, the *35S:amiR‐TSWV* construct (Figures 4a and S4) expressing the most effective anti‐TSWV amiRNA (amiR‐TSWV, with identical sequence and TS than syn‐tasiR‐TSWV‐1) selected from a previous functional screening (Carbonell *et al*., 2019) was also introduced in *S. lycopersicum*. Nine independent transgenic lines were generated, all of them with a phenotype indistinguishable from that of NTCs, and amiRNA accumulation in each line was analyzed by northern blot analysis of RNA preparations from apical leaves. Results showed that amiR‐TSWV accumulation was similar in seven of the nine lines, whereas two of the lines, *35S:amiR‐TSWV‐8* and *35S:amiR‐TSWV‐13*, accumulated considerably higher levels of amiR‐TSWV (Figure 4b).

Transgenic lines and NTCs were challenged with TSWV and analyzed for viral resistance/susceptibility as described above for syn‐tasiRNAs. Seven amiRNA lines showed symptoms or accumulated TSWV at 40 dpi, at least in one of the two plants inoculated, whereas only the two lines with higher amiR‐TSWV accumulation, *35S:amiR‐TSWV‐8* and *35S:amiR‐TSWV‐13*, were symptom‐ and virus‐free (Figure 4c,d; Table 2). These results confirm that there is a positive correlation between artificial sRNA accumulation and induced resistance level, and indicate that amiR‐TSWV is less effective than syn‐tasiR‐TSWV against TSWV in tomato plants. Finally, genomic TSWV RNA accumulation in representative amiRNA lines and NTCs measured at 40 dpi by RT‐qPCR (Figure S5) confirmed the results obtained by ELISA analysis. As observed for syn‐tasiR‐TSWV, amiR‐TSWV could be acting primarily through the endonucleolytic cleavage of viral RNAs, based on the comparison between TSWV RNA and protein levels in susceptible plants from lines *35S:amiR‐TSWV‐1* and *35S:amiR‐TSWV‐5* (Figure S5).

**Table 2:** Symptom and virus accumulation analyses in *Solanum lycopersicum* (tomato) transgenic plants expressing expressing antiviral amiRNAs infected with *Tomato spotted wilt virus* (TSWV)

Tomato transgenic line	Inoculated with TSWV	Analysis at 20 dpi		Analysis at 40 dpi	
		Symptomatic plants/total	DAS‐ELISA positive/total	Symptomatic plants/total	DAS‐ELISA positive/total
NTC	−	0/2	0/2	0/2	0/2
	+	2/2	2/2	2/2	2/2
*35S:amiR‐TSWV*					
1	+	0/2	0/2	2/2	2/2
3	+	0/2	1/2	1/2	1/2
5	+	0/2	0/2	1/2	1/2
8	+	0/2	0/2	0/2	0/2
10	+	2/2	2/2	2/2	2/2
11	+	0/2	1/2	2/2	2/2
12	+	0/2	0/2	2/2	2/2
13	+	0/2	0/2	2/2	0/2
14	+	0/2	0/2	2/2	2/2

DAS‐ELISA, double antibody sandwich enzyme‐linked immunosorbent assay; dpi, days post‐inoculation.

### Artificial sRNA accumulation does not explain the differences in antiviral activity between syn‐tasiR‐TSWV and amiR‐TSWV

**Figure 5:**
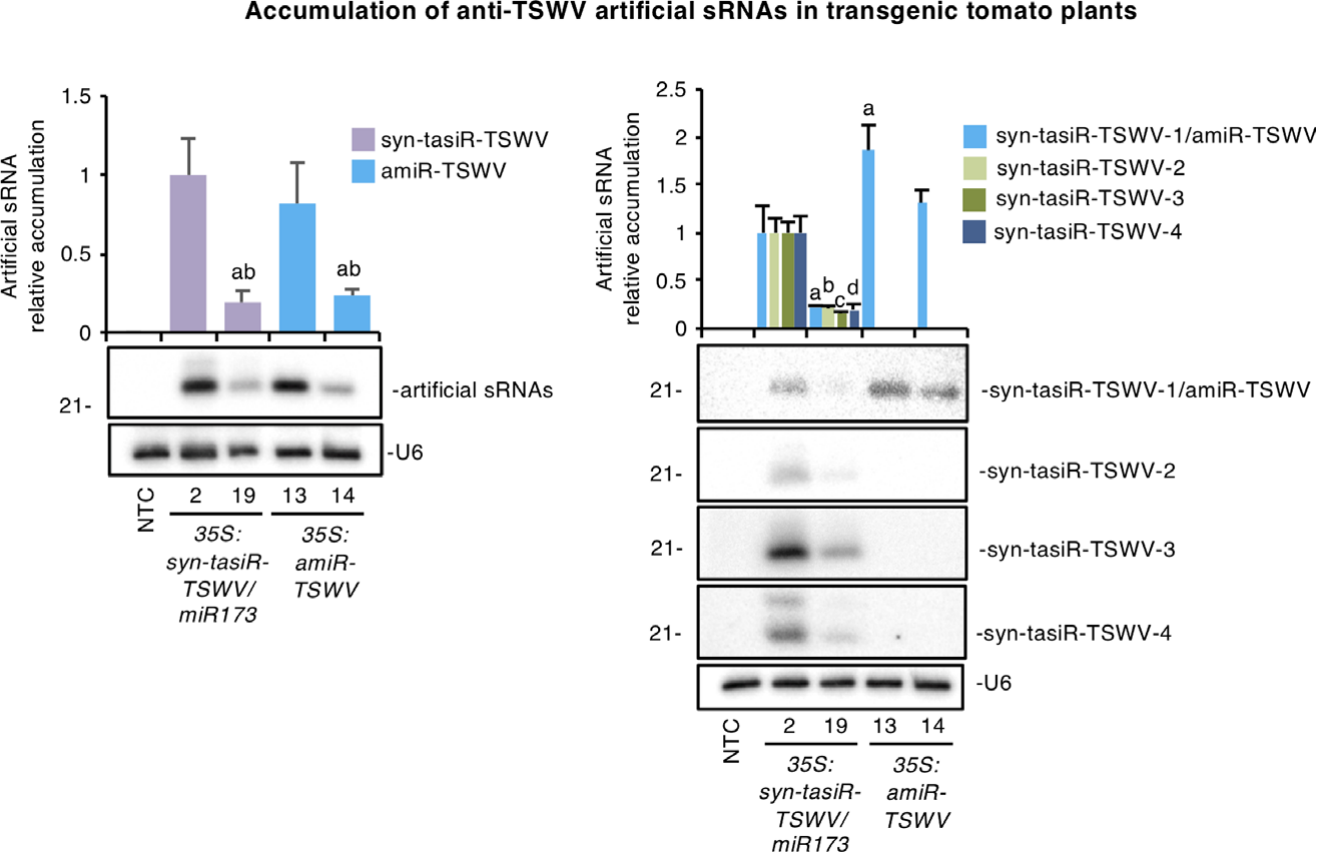
Comparative analysis of the accumulation of anti‐*Tomato spotted wilt virus* (anti‐TSWV) artificial small RNAs in transgenic tomato leaves. Left, northern blot detection of syn‐tasiR‐TSWV (syn‐tasiR‐TSWV‐1 + syn‐tasiR‐TSWV‐2 + syn‐tasiR‐TSWV‐3 + syn‐tasiR‐TSWV‐4) and amiR‐TSWV in RNA preparations from apical leaves of selected transgenic tomato lines and non‐transgenic controls. The graph at the top shows the mean (*n *=* *3) + standard deviation (SD) syn‐tasiR‐TSWV (purple) and amiR‐TSWV (blue) accumulation relative to that of the *35S:syn‐tasiR‐TSWV/MIR173‐A2* sample. Bars with the letter ‘a’ and ‘b’ are significantly different from that of samples *35S:syn‐tasiR‐TSWV/miR173‐A2* and *35S:amiR‐TSWV‐B1*, respectively (*P *<* *0.01 in pairwise Student's *t*‐test comparisons). Right, northern blot detection of individual syn‐tasiR‐TSWV species and of amiR‐TSWV in the same RNA preparations used in the left panel. The top graph shows the mean (*n *=* *3) + SD syn‐tasiR‐TSWV‐1/amiR‐TSWV (light blue), syn‐tasiR‐TSWV‐2 (light green), syn‐tasiR‐TSWV‐3 (dark green) and syn‐tasiR‐TSWV‐4 (dark blue) accumulation relative to syn‐tasiR‐TSWV‐1 accumulation in the *35S:syn‐tasiR‐TSWV/miR173‐A2* sample. Bars with the letters ‘a’, ‘b’, ‘c’ and ‘d’ are significantly different from that of syn‐tasiR‐TSWV‐1, syn‐tasiR‐TSWV‐2, syn‐tasiR‐TSWV‐3 and syn‐tasiR‐TSWV‐4, respectively, in sample *35S:syn‐tasiR‐TSWV/miR173‐A2* (*P *<* *0.01 in pairwise Student's *t*‐test comparisons). Other details are the same as in Figure 2(a).

To elucidate the reason(s) explaining the higher antiviral performance of syn‐tasiRNAs, we first compared the accumulation of syn‐tasiR‐TSWV and amiR‐TSWV in a line with high accumulation and in a line with low accumulation of each class (Figure 5). In particular, *35S:syn‐tasiR‐TSWV/miR173‐2* and *35S:amiR‐TSWV‐13* were selected as lines with high accumulations of syn‐tasiR‐TSWV and amiR‐TSWV, respectively, whereas *35S:syn‐tasiR‐TSWV/miR173‐19* and *35S:amiR‐TSWV‐14* were selected as lines with low accumulations of syn‐tasiR‐TSWV and amiR‐TSWV, respectively. Northern blot analysis of RNA preparations from apical leaves was performed with a cocktail of four probes to simultaneously detect the five artificial sRNAs (syn‐tasiR‐TSWV‐1/amiR‐TSWV + syn‐tasiR‐TSWV‐2 + syn‐tasiR‐TSWV‐3 + syn‐tasiR‐TSWV‐4). Blot analysis revealed that *35S:syn‐tasiR‐TSWV/miR173‐2* and *35S:amiR‐TSWV‐13* accumulated similar levels of artificial sRNAs (syn‐tasiR‐TSWV and amiR‐TSWV, respectively) (Figure 5, left). Similarly, *35S:syn‐tasiR‐TSWV/miR173‐19* and *35S:amiR‐TSWV‐14* accumulated similar levels of syn‐tasiR‐TSWV and amiR‐TSWV, respectively (Figure 5, left). These results suggest that the higher antiviral activity of the majority of the syn‐tasiRNA lines is not the consequence of a higher accumulation of syn‐tasiR‐TSWV compared with amiR‐TSWV.

To check the specific accumulation of each of the four syn‐tasiRNA sequences expressed from these two syn‐tasiRNA lines, the same RNA preparations described above were also analyzed by northern blot, but in this case with individual probes to detect syn‐tasiR‐TSWV‐1/amiR‐TSWV, syn‐tasiR‐TSWV‐2, syn‐tasiR‐TSWV‐3 or syn‐tasiR‐TSWV‐4, independently. Blot analyses showed that syn‐tasiR‐TSWV‐3 accumulated to a higher level, followed by syn‐tasiR‐TSWV‐4, syn‐tasiR‐TSWV‐1 and syn‐tasiR‐TSWV‐2 (Figure 5, right). It is important to note that although each blot analysis was performed in parallel and using the same quantity of each probe, differences in hybridization efficiency between individual probes cannot be ruled out and may influence the level of signal detected. Interestingly, syn‐tasiR‐TSWV‐1 accumulation was lower than that of amiR‐TSWV in both the high‐ and low‐accumulation lines (Figure 5, right). Altogether, these results suggest that the higher antiviral activity of syn‐tasiR‐TSWV lines may be a result of the combined effect of the four syn‐tasiRNAs that are expressed.

### The simultaneous targeting of multiple target sites by syn‐tasiRNAs limits the ability of TSWV to introduce target site mutations and overcome resistance

**Figure 6:**
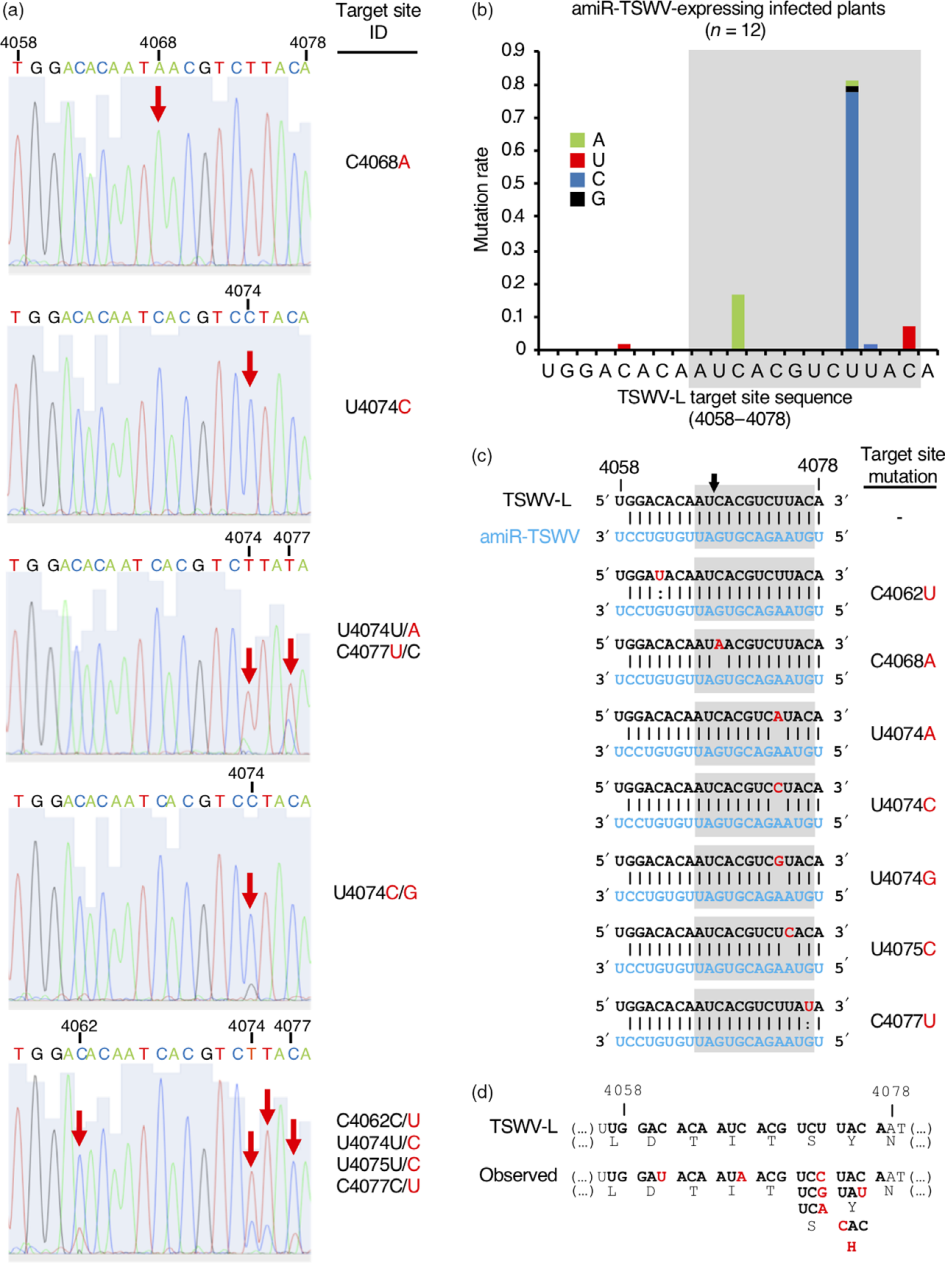
Target site (TS) analysis in *Tomato spotted wilt virus* (TSWV)‐infected amiR‐TSWV lines. (a) Electropherograms derived from sequencing TSWV progeny, including the TS region only. One example of each TS class is shown, with positions including significant nucleotide mutations highlighted with a red arrow. TS IDs include wild‐type nucleotides and coordinates in black, mutations in red, and are named using the nucleotide position preceded and followed by the wild‐type and mutated nucleotides, respectively. When a position contains more than one significant peak, the corresponding TS ID includes the most frequent nucleotide first, and then the nucleotide position. (b) Bar graph representing the mutation rate for each TS position corresponding to nucleotides 4058–4078 of TSWV segment L. The identity of the mutations is indicated in the color legend inserted in the graph. Wild‐type nucleotides are in black. Data were collected after sequencing TSWV progeny from all 12 infected amiRNA plants. (c) Predicted amiRNA/TS base pairing configurations including individual target site mutations. Details are as described above and as in Figure 1(a). (d) Effect on the amino acid composition of TSWV RdRP of each individual nucleotide substitution observed in TSs corresponding to positions 4058–4078 of TSWV segment L. Wild‐type nucleotides and amino acids are in black; mutated nucleotides and amino acids are in red. Top, nucleotide and amino acids of wild‐type TSWV. Bottom, observed nucleotides and amino acids in viral progenies sequenced from amiR‐TSWV‐expressing infected plants.

Next, TS sequences from viral progenies of TSWV‐infected transgenic plants were analyzed by RT‐PCR followed by Sanger sequencing. Interestingly, none of the four different TSs recovered from the four infected syn‐tasiRNA‐expressing plants contained mutations with respect to the TSWV‐LL‐N.05 consensus sequence (Table 3). In contrast, all TSs recovered from infected amiRNA lines presented nucleotide variability in one (C4068A, U4074C) or more positions (U4074C/G, U4074U/A‐C4077U/C and C4062C/U‐U4074U/C‐U4075U/C‐C4077C/U) (Table 3), as observed in the electropherograms derived from sequencing (Figure 6a). The most frequent TS modification was U4074C (7/12), followed by C4068A (2/12), U4074C/G (1/12), U4074U/A‐C4077U/C (1/12) and C4062C/U‐U4074U/C‐U4075U/C‐C4077C/U (1/12) (Table 3). Next, the mutation rate of each TS nucleotide position was estimated and plotted (Figure 6b). From the corresponding graph, we conclude that: (i) position 4074 has the highest mutation rate (above 81%), and was mutated to a C, a G or an A in 77.6, 1.8 and 1.6% of cases, respectively; and (ii) that all mutations except C4062U are located in the mismatch‐sensitive region of the TS (Figure 6b). Interestingly, the analysis of the effect of each individual mutation in the predicted amiRNA/TS base pairing configurations reveals that the majority (5/7) of the observed substitutions (C4068A, U4074A, U4074C, U4074G and U4075C) lead to mismatches in the mismatch‐sensitive region (Figure 6c). Only the C4062U and C4077U substitutions lead to G:U base pairs (Figure 6c). Finally, the effect of each individual nucleotide substitution on the amino acid composition of TSWV RdRP was analyzed (Figure 6d). Only the U4075C mutation leads to an amino acid change [from a tyrosine (Y) to a histidine (H)], whereas all the rest of the substitutions are silent (Figure 6d), which indicates that nucleotide substitutions introduced by TSWV at the amiR‐TSWV TS generally preserve the amino acid composition of the corresponding protein. In summary, all of these results indicate that the infection of amiRNA lines is characterized by the ability of TSWV to mutate the amiRNA TS while preserving its capacity to spread systemically.

**Table 3:** Sequence analysis of artificial sRNA target sites in TSWV‐infected tomato transgenic plants^a^

Transgenic line	Artificial sRNA	Target site						
		TSWV segment	Coordinates	ID	Frequency	Position	Primary sequence	Secondary sequence
*35S:syn‐tasiR‐TSWV/MIR173*	syn‐tasiR‐TSWV‐1	L	4058‐4078	WT	4/4	–	–	–
	syn‐tasiR‐TSWV‐2	M	4494‐4514	WT	4/4	–	–	–
	syn‐tasiR‐TSWV‐3	M	507‐527	WT	4/4	–	–	–
	syn‐tasiR‐TSWV‐4	L	771‐791	WT	4/4	–	–	–
*35S:amiR‐TSWV*	amiR‐TSWV	L	4058‐4078	WT	0/12	–	–	–
				C4068A	2/12	4068	A	–
				U4074C	7/12	4074	C	–
				U4074C/G	1/12	4074	C	G
				U4074U/A	1/12	4074	U	A
				C4077U/C		4077	U	C
				C4062C/U	1/12	4062	C	U
				U4074U/C		4074	U	C
				U4075U/C		4075	U	C
				C4077C/U		4077	C	U

a Observed mutations are in red.

## Discussion

### Resistance to TSWV in *S. lycopersicum* mediated by syn‐tasiRNAs

The economic impact of TSWV has increased during the last decades, causing high yield losses in a variety of plant species, including economically important crops such as tomato and pepper (Turina *et al*., 2016). Recently, highly specific approaches based on the expression of artificial sRNA transgenes have been reported in model plants such as *N. benthamiana* or *Nicotiana tabacum* (Mitter *et al*., 2016; Carbonell *et al*., 2019). Here, we aimed to systematically analyze and compare the anti‐TSWV resistance induced by the expression of amiRNAs and syn‐tasiRNAs in the natural hostof TSWV, *S. lycopersicum*. Results indicate that both artificial sRNA‐based strategies induced resistance; however, a higher proportion of syn‐tasiR‐TSWV lines was resistant, whereas most of amiRNA lines were susceptible. Interestingly, all infected amiRNA lines accumulated TSWV mutants with altered TS sequences that escaped amiR‐TSWV targeting. All in all, our results suggest that syn‐tasiRNAs may be the preferred artificial sRNA‐based tool to engineer more efficient, durable and specific anti‐TSWV resistance in tomato plants.

### Subinhibitory accumulation of amiR‐TSWV allows virus evasion of the antiviral resistance through the accumulation of TS mutations

**Figure 7:**
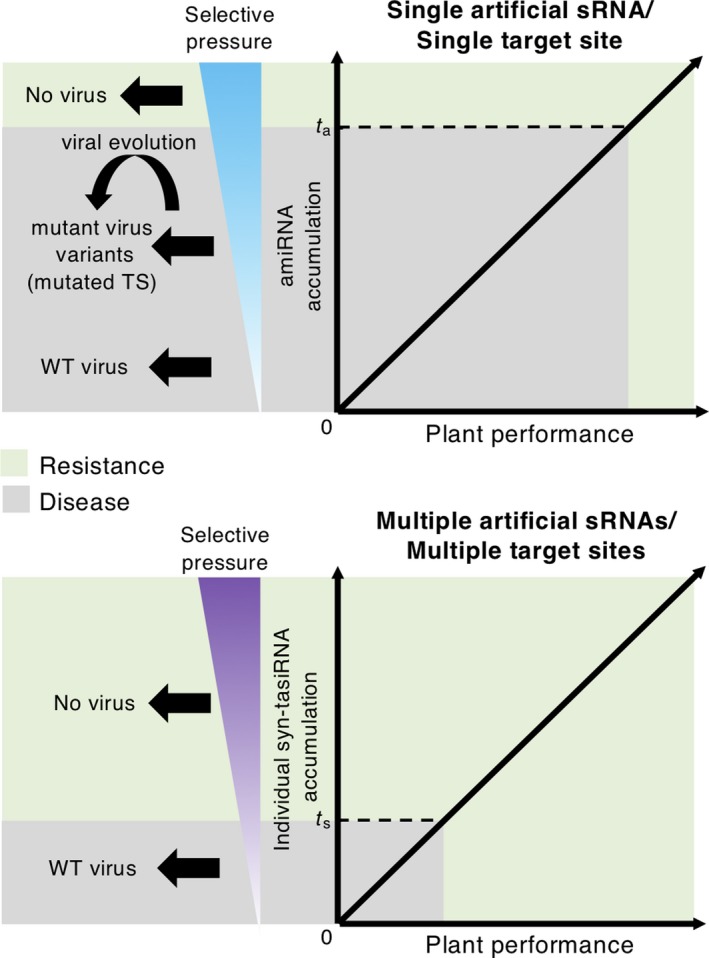
Model describing the differential effects of amiR‐TSWV and syn‐tasiR‐TSWV accumulation on virus replication and disease resistance. Top, graph representing a theoretical positive (linear) correlation between amiRNA accumulation and plant performance. *t*_a_ represents the amiRNA accumulation threshold, above which plants are resistant to TSWV. At subinhibitory amiRNA concentrations (most frequent scenario), TSWV can introduce TS mutations that may help TSWV to escape from amiRNA targeting. Bottom, graph representing a theoretical positive (linear) correlation between syn‐tasiRNA accumulation and plant performance. *t*_s_ represents the syn‐tasiRNA accumulation threshold, above which plants are resistant to TSWV. At subinhibitory syn‐tasiRNA concentrations (the less frequent scenario), TSWV does not mutate to induce disease. Note that *t*_s_ < *t*_a_, thus indicating that resistance is easier to obtain through syn‐tasiRNAs.

**Figure 8:**
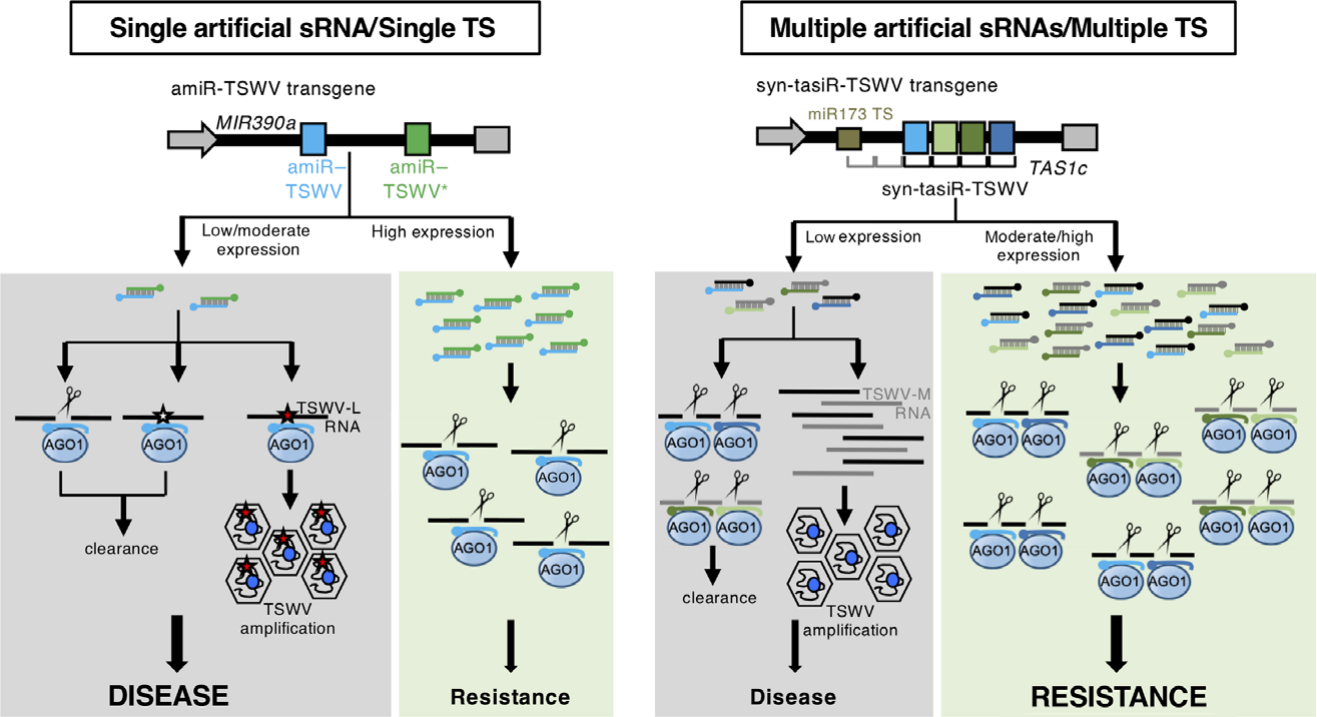
Model comparing the antiviral effects of anti‐*Tomato spotted wilt virus* (anti‐TSWV) artificial sRNA constructs. Left, antiviral effects of single targeting with amiR‐TSWV. At subinhibitory artificial sRNA concentrations (low amiRNA accumulation, observed in the majority of amiRNA lines), TSWV replication can generate non‐deleterious mutations (red stars) in TSs that may affect the base‐paring between amiRNA and target RNA nucleotides. As a result, amiR‐TSWV activity may be inhibited, which can favor TSWV escape to induce disease. At the same time, it is also possible that TSs in viral RNAs accumulate deleterious mutations (white stars), or no mutations at all. In both cases, viral RNAs will be ultimately degraded. At inhibitory artificial sRNA concentrations (high amiRNA accumulation, observed in the minority of amiRNA lines), amiR‐TSWV efficiently degrades TSWV‐L RNAs though its association with AGO1, and induces resistance. Right, antiviral effects of multi‐targeting with syn‐tasiR‐TSWV. At subinhibitory concentrations (low syn‐tasiRNA accumulation, observed in the minority of syn‐tasiRNA lines), syn‐tasiR‐TSWV/AGO1‐mediated silencing of viral RNAs is insufficient to prevent viral replication, spread and disease. At inhibitory concentrations (high syn‐tasiRNA accumulation, observed in the majority of syn‐tasiRNA lines), the four syn‐tasiR‐TSWV species may efficiently degrade TSWV‐L and TSWV‐M RNAs through their association with AGO1 to induce resistance.

The majority of amiRNA‐expressing lines were susceptible, with all susceptible lines accumulating moderate levels of amiR‐TSWV compared with the two resistant amiRNA lines (Figure 4a). The higher amiR‐TSWV accumulation in resistant lines is not surprising, as it is well known that there is a positive correlation between the accumulation of an antiviral amiRNA and the degree of induced resistance (Niu *et al*., 2006; Qu *et al*., 2007; Kung *et al*., 2012). Thus, our results suggest the existence of a threshold level of amiRNA accumulation (*t*_a_) below which the virus targeting by amiR‐TSWV is inefficient and cannot impede viral replication and spread (Figure 7, top). Interestingly, all infected amiRNA lines accumulated viral progenies with TS mutations. These mutations, likely derived from viral RdRP error‐prone activity during TSWV replication, generally disrupted the base pairing between amiR‐TSWV and TSWV, which most likely helped TSWV to escape from amiR‐TSWV targeting (Figure 8, left). As proposed before, such TS mutations typically emerge when, although at a subinhibitory concentration, the selective pressure imposed by the amiRNA is sufficiently high (Lafforgue *et al*., 2011). It is also possible that other mutations that do not affect amiR‐TSWV binding to TSs, that do not favor TSWV replication or that critically modify RdRP amino acid composition were transiently generated but not fixed in TSWV progenies because of their deleterious nature (Figure 8, left). In any case, the absence of viral progenies with the wild‐type sequence in infected amiRNA lines suggests that amiR‐TSWV accumulation in these lines was not particularly low, but instead was moderate. Hence, the main reason explaining the lack of resistance in amiRNA lines accumulating amiR‐TSWV at subinhibitory concentrations (below *t*_a_) seems to be the emergence of these resistant TSWV mutants with altered TSs. These viral escapees with increased fitness are able to replicate and move despite the presence of antiviral amiRNAs, whereas wild‐type genomes are productively targeted (Figure 8, left). A similar scenario was proposed for describing the emergence of resistant mutants carrying amiRNA TSs in coding or non‐coding sequences in several plant viruses (Simon‐Mateo and Garcia, 2006; Lin *et al*., 2009; Lafforgue *et al*., 2011). It is also possible that some TS mutations do not impede amiR‐TSWV‐mediated degradation and thus do not confer any particular advantage to TSWV (Figure 8, left). This might be the case of the C4062U mutation that generates a G:U wobble base pair outside the mismatch‐sensitive region (Figure 6c).

The TS mutations observed were all nucleotide substitutions: 70% were transitions (all U↔C) and 30% were transversions (10% C→A; 10% U→G; 10% U→A), which generally affected the base‐pairing between amiRNA and TSWV nucleotides in the mismatch‐sensitive region but did not alter the amino acid sequence of TSWV RdRP (Figure 6c,d). The following considerations arose after analyzing these results. First, the excess of transition over transversion mutations is not surprising, as this has already been observed in TSs located in coding regions of *Turnip mosaic virus* (TuMV) subject to amiRNA selective pressure (Lafforgue *et al*., 2011). More generally, it has been proposed that viral coding regions accumulate more transition than transversion mutations, not only because biochemical transitions are more likely to occur, but also possibly through the action of plant RNA‐editing cytidine deaminases that could induce transition mutations in single‐stranded regions of viral genomes (Lafforgue *et al*., 2011), a well‐described phenomenon for *Human immunodeficiency virus* (HIV) and other retroviruses (Bishop *et al*., 2004; Cullen, 2006). This situation where only nucleotide substitutions are observed, however, differs when TSs are not imbedded into viral coding sequences, as mutant variants including nucleotide deletions or insertions are recovered (Simon‐Mateo and Garcia, 2006; Lin *et al*., 2009). Second, the clustering of observed TS mutations in the mismatch‐sensitive region should not be shocking, as it has already been reported for TSs included in several plant viruses (Simon‐Mateo and Garcia, 2006; Lin *et al*., 2009; Lafforgue *et al*., 2011; Martinez *et al*., 2012). In plants, a high degree of base pairing between an sRNA and its target RNA is required for AGO‐mediated slicing (Liu *et al*., 2014), and it is known that mismatches between positions 2–13 are not well tolerated (Schwab *et al*., 2005; Fahlgren and Carrington, 2010). In particular, imperfect pairing with central mismatches in sRNA‐target hybrids favors translation repression over slicing (Brodersen *et al*., 2008), an inefficient process requiring stoichiometric quantities of amiRNA that may allow sufficient viral replication for escaping from amiRNA action. Here, the most frequent mutation in TSs from the region 4058–4078 of TSWV segment L is U4074C, which was observed in 10 of the 12 independent progenies. This mutation, close to the 5′ end of amiR‐TSWV, may affect target slicing, whereas the second most frequent mutation, C4068A, inducing a central mismatch may trigger translation repression of TSWV‐L RNA. In any case, RNAi viral escapees with altered TS sequences have also been observed in human viruses such as *polio virus*,* Hepatitis C virus*, HIV and *Japanese encephalitis virus* (JEV), and also in animal viruses such as the model morbillivirus *Peste des petits ruminants virus* (PPRV) (Presloid and Novella, 2015). Still, resistance mutations may not always be located in TSs, suggesting that different mechanisms can be used to escape RNAi (Berkhout and Das, 2012; Shah *et al*., 2012). Third, despite that the selected TSs were intentionally included in sequence‐conserved regions for broadening the induced resistance (Carbonell *et al*., 2019), TSWV variants with mutations in the 4058–4078 region of segment L emerged in infected amiRNA lines. Interestingly, none of the observed mutations except C4062U were found in natural TSWV sequences deposited in databases (Figure S6), strongly suggesting that they were not present in the initial inoculum, but rather that they were generated and selected during viral replication under amiRNA selective pressure. And fourth, 90% of the mutations were synonymous, did not affect the coding sequence of TSWV RdRP and mapped to the third nucleotide position of the corresponding codon (Figure 6d). Only the U4075C mutation leads to an amino acid change, from a tyrosine to a histidine. Still, the particularly short height of the cytosine peak compared with that of the wild‐type thymine in position 4075 of the corresponding electropherogram may indicate that this change was not yet fixed, or that it belonged to a virus mutant that was being cleared. Altogether, our results indicate that TS mutations that were preferentially selected probably interfered with amiR‐TSWV function, and resulted in an advantage for TSWV replication or movement while preserving the amino acid identity of the RdRP.

### Molecular basis of enhanced antiviral resistance through multi‐targeting of viral RNAs with syn‐tasiRNAs

In contrast to amiRNA lines, the majority of syn‐tasiRNA lines were resistant to TSWV. Only the two lines with lower accumulations of syn‐tasiR‐TSWV were susceptible. This result suggests a positive correlation between the accumulation of syn‐tasiR‐TSWV and the degree of antiviral resistance induced, and the existence of a syn‐tasiRNA accumulation threshold (*t*_s_) below which syn‐tasiRNA‐mediated targeting of TSWV RNAs is inefficient and disease is triggered (Figure 7, bottom). This last scenario is rather rare in our study, as only a low proportion of syn‐tasiRNA lines was infected. Interestingly, no TS mutations were observed in TSWV progeny from syn‐tasiRNA‐infected plants (Figure 8, right). Perhaps in these lines syn‐tasiR‐TSWV accumulation was particularly low, and the selective pressure exerted by syn‐tasiR‐TSWV was insufficient to trigger the accumulation of TS mutations. Or perhaps TSWV mutants accumulating mutations in at least one but not all TSs were ultimately degraded by the action of the syn‐tasiRNA(s), the TS(s) of which did not mutate. In any case, based on the quantitative analyses of syn‐tasiR‐TSWV and amiR‐TSWV accumulation (Figures 3a, 4b and 5), *t*_s_ might be lower than *t*_a_ (Figure 7, bottom), as several syn‐tasiRNA lines with rather low accumulations of syn‐tasiR‐TSWV (e.g. lines *35S:syn‐tasiR‐TSWV/miR173‐A7*, ‐*A11* or ‐*B18*; Figure 3a) are resistant. This requirement for lower syn‐tasiR‐TSWV concentrations to gain resistance may be explained by the combined silencing effect of the four individual syn‐tasiR‐TSWV species that makes it less probable that each different TS holds the mutation(s) necessary to fully escape each syn‐tasiR‐TSWV. In several studies on human‐infecting viruses, the use of siRNA pools produced from short or long hpRNAs targeting HIV (Gitlin *et al*., 2005; ter Brake *et al*., 2006; von Eije *et al*., 2008) and JEV (Nishitsuji *et al*., 2006) was more effective in protecting against viral infection than single siRNAs, although escape mutants were found. Moreover, these approaches may lead to off‐target effects, despite the siRNA species in the pool being present at very low concentrations. In our study, although each of the four syn‐tasiR‐TSWV sequences were designed with p‐sams (Fahlgren *et al*., 2016) to be highly specific with no predicted off targets in *S. lycopersicum* (Carbonell *et al*., 2019), the possibility that syn‐tasiR‐TSWV induces the accidental targeting of endogenous transcripts cannot be completely ruled out. This scenario seems unlikely, as has been shown for four other p‐sams‐based amiRNAs in *Brachypodium distachyon* through genome‐wide transcriptome profiling combined with 5′‐RLM‐RACE analyses (Carbonell *et al*., 2015).

### Syn‐tasiRNAs as an emerging antiviral tool for crop protection

The multi‐targeting of plant viruses through artificial sRNAs was first achieved with transgenes expressing multiple amiRNAs from different precursors *in* *cis* (Ai *et al*., 2011; Kung *et al*., 2012) or *in* *trans* (Lafforgue *et al*., 2013), or from a single polycistronic precursor (Fahim *et al*., 2012; Kis *et al*., 2016), and more recently with transgenes expressing multiple syn‐tasiRNAs from *TAS1c* (Carbonell and Daros, 2017; Carbonell *et al*., 2019) or *TAS3a* precursors (Chen *et al*., 2016), although neither the durability of the resistance nor the comparison with single targeting was systematically studied. Here, we analyzed the anti‐TSWV effects of two artificial sRNA constructs in parallel, one expressing an amiRNA and another expressing four syn‐tasiRNAs, stably expressed in *S. lycopersicum*. Although the two constructs were previously described to be similarly effective against TSWV when transiently assayed in *N. benthamiana* (Carbonell *et al*., 2019), syn‐tasiRNAs were more effective than amiRNA against TSWV when stably expressed in *S. lycopersicum* plants. Now, we suspect that we could not observe differences in amiR‐TSWV and syn‐tasiR‐TSWV efficacies in our previous work (Carbonell *et al*., 2019), most likely because amiR‐TSWV was not present at subinhibitory concentrations during the overexpression analysis in *N. benthamiana* leaves.

In recent years, several bacterial CRISPR/Cas systems such as CRISPR/Cas9, CRISPR/Cas13 and CRISPR/Cas14 have been used to edit the DNA, RNA and single‐stranded DNA, respectively, of several plant viruses, through a single guide RNA (sgRNA) to induce antiviral resistance (for recent reviews, see Chaudhary, 2018; Khan *et al*., 2018, 2019; Mahas and Mahfouz, 2018); however, these systems, particularly CRISPR/Cas9, have major drawbacks. First, at least for some plant DNA viruses (Ali *et al*., 2016; Tashkandi *et al*., 2018; Mehta *et al*., 2019), as well as for HIV (Wang *et al*., 2016a,b; Yoder and Bundschuh, 2016), the emergence of viral escapees has also been reported. Indeed, a major risk of the CRISPR/Cas9‐based antiviral approach is the possible generation of virus variants resistant to Cas9 cleavage, and in the worst‐case scenario, to altered viruses with greater pathogenicity. As for syn‐tasiRNAs, the use of more sophisticated CRISPR/Cas systems allowing the multiplexing of multiple sgRNAs in a single construct should reduce the emergence of viral escapees, as proposed before (Mahas and Mahfouz, 2018). Second, the possibility of permanent, potentially deleterious off‐target changes to the host genome exist, especially for CRISPR/Cas9. Therefore, despite the big impact of recent antiviral CRISPR/Cas systems, we anticipate that the syn‐tasiRNA approach will remain an attractive antiviral tool for its unique features of multi‐targeting and high specificity (Carbonell, 2019b), as well as for the availability of high‐throughput cloning strategies and automated design tools for the simple generation of syn‐tasiRNA constructs (Carbonell *et al*., 2014; Carbonell, 2019a).

## Experimental procedures

### Plant materials and growth conditions

*Nicotiana benthamiana* and *S. lycopersicum* cv. Moneymaker *Tm2* ^*2*^ plants were grown in a growth chamber at 25°C with a 12 h‐light/12 h‐dark photoperiod.

### DNA constructs

*35S:GUS*,* 35S:MIR173*,* 35S:amiR‐TSWV*,* 35S:syn‐tasiR‐TSWV* and *35S:syn‐tasiR‐GUS* were reported previously (Montgomery *et al*., 2008b; Carbonell *et al*., 2019). The *35S‐MIR173‐Tnos* cassette was PCR‐amplified from the *35S:MIR173* construct using oligonucleotides AC‐14 and AC‐15 (Table S1), gel purified, and assembled into *Eco*RI‐digested and gel‐purified *35S:syn‐tasiR‐PSTVd* or *35S:syn‐tasiR‐GUS* DNAs in the presence of NEBuilder HiFi DNA Assembly Master Mix (New England Biolabs, https://www.neb.com) to generate *35S:syn‐tasiR‐TSWV/miR173* and *35S:syn‐tasiR‐GUS/miR173*, respectively. The sequences of all artificial sRNA‐generating precursors used in this study are listed in Appendix S1.

### Generation of tomato transgenic plants

*Agrobacterium tumefaciens* LBA4404 transformed with *35S:syn‐tasiR‐TSWV/miR173* or *35S:syn‐tasiR‐GUS/miR173* were co‐cultured with tomato cotyledons. Explant preparation, selection and regeneration were performed as described by Ellul *et al*. (2003). Transformants were selected in hygromycin‐containing medium, and then propagated in soil for the infection studies. Non‐transgenic *in vitro*‐regenerated tomato plants, obtained in parallel with the transgenic plants, were used as controls for the analyses.

### Transient expression of constructs and virus infection assays

Agroinfiltration of constructs in *N. benthamiana* leaves was performed as described by Llave *et al*. (2002) and Cuperus *et al*. (2010), using strain GV3101 of *A. tumefaciens*. Virus infection assays with TSWV LL‐N.05 isolate (Debreczeni *et al*., 2015) were performed as described before (Carbonell and Daros, 2019; Carbonell *et al*., 2019).

### Small RNA gel northern blot assays

Total RNA from *S. lycopersicum* or *N. benthamiana* leaves was isolated using TRIzol reagent (ThermoFisher Scientific, https://www.thermofisher.com) followed by chloroform extraction and isopropanol precipitation as described by Cuperus *et al*. (2010). RNA gel blot assays including RNA separation in 17% polyacrylamide gels containing 0.5 × Tris/Borate/EDTA and 7 m urea, RNA transfer to positively charged nylon membrane, probe synthesis using [γ‐^32^P]ATP (PerkinElmer, https://www.perkinelmer.com) and T4 polynucleotide kinase (ThermoFisher Scientific) were performed as described by Montgomery *et al*. (2008a) and Cuperus *et al*. (2010). A Typhoon Trio Variable Mode Imager System (Amersham Biosciences, now GE Healthcare Life Sciences, https://www.gelifesciences.com) was used to produce digital images from radioactive membranes. imagequant 5.2 (Molecular Dynamics) was used for the quantification of hybridization bands. The DNA oligonucleotides used as probes for sRNA blots are listed in Table S1.

### DAS‐ELISA assays

The accumulation of TSWV was analyzed by double antibody sandwich enzyme‐linked immunosorbent assay (DAS‐ELISA) using the TSWV Complete kit (Bioreba, http://www.bioreba.ch), as described by Carbonell *et al*. (2019). DAS‐ELISA analyses were performed in extracts obtained from 1 g of apical *N. benthamiana* (collected at 20 dpi) or *S. lycopersicum* (collected at 20 and 40 dpi) leaves. In DAS‐ELISA positive (infected) samples, the absorbance was higher than three times the average absorbance of the samples from mock‐inoculated controls.

### RT‐qPCR

RT‐qPCR analysis was performed in the same tissues that were analyzed by ELISA, essentially as described by Carbonell *et al*. (2014). Briefly, total RNA from *S. lycopersicum* leaves was isolated using TRIzol reagent (ThermoFisher Scientific) as described above. DNaseI‐treated total RNA (2 μg) served to produce first‐strand complementary DNA (cDNA) using the SuperScript IV system (ThermoFisher Scientific), and oligonucleotides dT or AC‐203 for endogenous or TSWV‐L transcript quantification, respectively. qPCR was performed on optical 96‐well plates in the 7500 Fast Real‐Time PCR System (Applied Biosystems, now ThermoFisher Scientific) using the following program: 20 s at 50°C and 10 min at 95°C, followed by 40 cycles of 95°C for 15 s and 60°C for 1 min, with an additional melt‐curve stage consisting of 15 s at 95°C, 1 min at 60°C, and 15 s at 95°C. The 20‐μl reaction mixture contained 10 μl of 2 X Maxima SYBR Green/ROX qPCR Master Mix (ThermoFisher Scientific), 2 μl of diluted cDNA (1:5), and 300 nm of each gene‐specific primer. Primers used for RT‐qPCR are listed in Table S1. Target mRNA expression levels were calculated relative to two *S. lycopersicum* reference genes [actin (*Tom41*) and *Elongation Factor 1α* (*eEF1α*)] using the delta delta cycle threshold (ΔΔ*C*_*t*_) comparative method (Applied Biosystems, now ThermoFisher Scientific) of the 7500 fast software 2.0.4 (Applied Biosystems). Two independent biological replicates, and two technical replicates for each biological replicate were analyzed.

### Sequence analysis of target sites in TSWV RNAs

The TS sequences from viral progeny of TSWV‐infected transgenic plants were analyzed by RT‐PCR followed by Sanger sequencing. cDNA was obtained from 1–5 μg of total RNA from apical *S. lycopersicum* leaves using RevertAid reverse transcriptase (RT; ThermoFisher Scientific). Briefly, for each sample, a mixture including the total RNA and 5 pmol of oligonucleotide AC‐203 or AC‐207 (for TSs in segments L and M, respectively) (Table S1) was incubated for 1.5 min at 98°C, and then transferred to ice. A 3.5‐μl mixture including 2 μl of RT buffer, 1 μl of 10 mm dNTPs, 0.25 μl of RiboLock RNase inhibitor (40 U μl^−1^; ThermoFisher Scientific) and 0.25 μl of RevertAid (200 U μl^−1^; ThermoFisher Scientific) was added to each sample. Samples were incubated for 45 min at 42°C, 10 min at 50°C, 5 min at 60°C and 15 min at 70°C. PCR to amplify TS fragments from segment L or segment M (604 bp or 537 bp, respectively) was performed using oligonucleotide pairs AC‐205/AC‐206 or AC‐209/AC‐210 (Table S1), respectively, and Phusion DNA polymerase (ThermoFisher Scientific) with the following program: 30 s at 98°C; 32 cycles of 98°C for 30 s, 50°C for 30 s, 72°C for 30 s; 10 min at 72°C. PCR products were analyzed by 2% agarose gel electrophoresis, and products of the expected size were excised from the gel and sequenced.

### Estimation of the mutation rate of target site positions

The mutation rate of each nucleotide position in TSs located in fragment L (positions 4058–4078) and recovered from infected amiRNA lines was estimated. Sequencing electropherograms were analyzed to identify the presence of single or multiple peaks in each TS position. In TS positions containing multiple peaks, peaks with >15% of the size of the predominant peak were included in the calculation of the mutation rate. In these cases, the relative size of each peak was estimated and used in the calculation of the mutation rate of the corresponding position.

## Data statement

All data relating to this manuscript can be found within the manuscript and its supplementary files.

## Conflict of interest

The authors declare no conflicts of interest.

## Author contributions

PL generated the tomato transgenic lines and revised the text. JAD analyzed the data and revised the text. AC designed the project, performed the experiments, analyzed the data and wrote the article.

## Supporting information


**Figure S1.** Diagram of the complete *35S:syn‐tasiR‐TSWV/miR173* plasmid.

**Figure S2.** Diagram of the *35S:syn‐tasiR‐GUS/miR173* construct.

**Figure S3.** Relative TSWV‐L RNA accumulation at 40 dpi in selected syn‐tasiRNA lines.

**Figure S4.** Diagram of the complete *35S:amiR‐TSWV* plasmid.

**Figure S5.** Relative TSWV‐L RNA accumulation at 40 dpi in selected amiRNA lines.

**Figure S6** **.** Multiple alignment of all sequences corresponding to TSWV segment L found in databases.

**Table S1.** Name, sequence and use of DNA oligonucleotides.

**Table S2.** Summary of results obtained from symptom and DAS‐ELISA analyses (upper non‐inoculated tissues) in *Nicotiana benthamiana* bioassays.

**Appendix S1.** DNA sequences in FASTA format of all artificial small RNA generating precursors used in this study.
