# Supplementary material for: Multi‐targeting of viral RNAs with synthetic trans‐acting small interfering RNAs enhances plant antiviral resistance
Source: Plant J. 2019 Sep 16;100(4):720–37. doi: 10.1111/tpj.14466 (PMC6899541; doi:10.1111/tpj.14466)
Supplement: Supplementary file 1 — Figure S1. Diagram of the complete 35S:syn‐tasiR‐TSWV/miR173 plasmid. [file TPJ-100-720-s001.pdf]

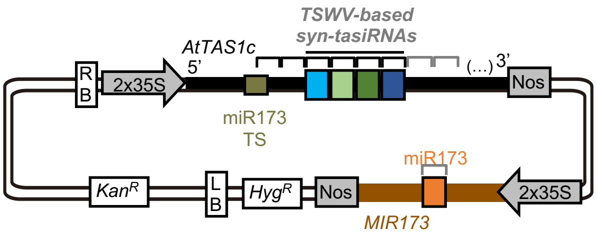

35S:syn-tasiR-TSWV/miR173

Figure S1. Diagram of the complete *35S:syn-tasiR-TSWV/miR173* plasmid. Approximate locations of T-DNA right and left borders (RB and LB, respectively) as well as bacterial and plant antibiotic resistance genes (kanamycin and hygromycin, respectively) are indicated. Other details are described in Figure 1a.
