# Supplementary material for: Multi‐targeting of viral RNAs with synthetic trans‐acting small interfering RNAs enhances plant antiviral resistance
Source: Plant J. 2019 Sep 16;100(4):720–37. doi: 10.1111/tpj.14466 (PMC6899541; doi:10.1111/tpj.14466)
Supplement: Supplementary file 2 — Figure S2. Diagram of the 35S:syn‐tasiR‐GUS/miR173 construct. [file TPJ-100-720-s002.pdf]

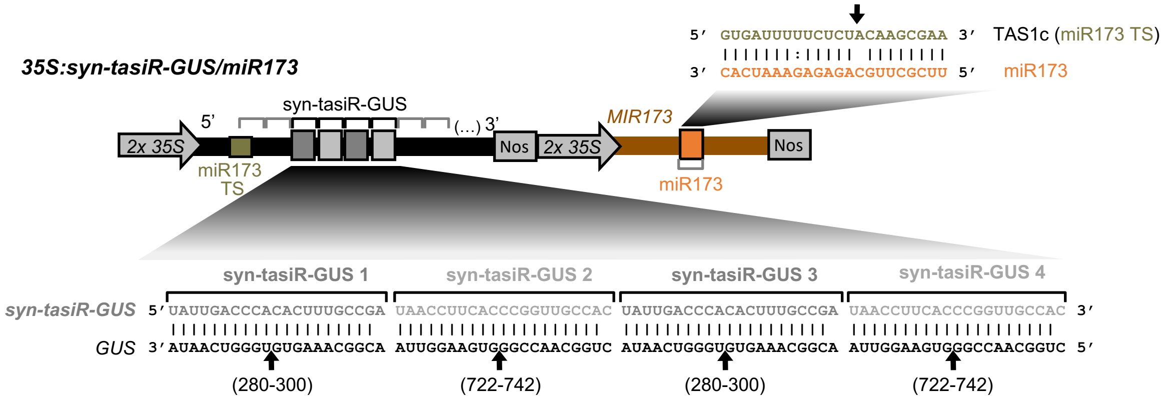

Figure S2. Diagram of the *35S:syn-tasiR-GUS/miR173* construct. *TAS1c* precursor from *A. thaliana* (in black) was engineered to produce four anti-GUS syn-tasiRNAs (in light or dark greyboxes). Base pairing between syn-tasiRNA and TSWV nucleotides (in grey and black, respectively) is shown. Specific cleavage sites in target sites located in GUS RNAs are indicated with black arrows, with TS coordinates indicated in brackets. Other details are described in Figure 1a.
