# Supplementary material for: Multi‐targeting of viral RNAs with synthetic trans‐acting small interfering RNAs enhances plant antiviral resistance
Source: Plant J. 2019 Sep 16;100(4):720–37. doi: 10.1111/tpj.14466 (PMC6899541; doi:10.1111/tpj.14466)
Supplement: Supplementary file 3 — Figure S3. Relative TSWV‐L RNA accumulation at 40 dpi in selected syn‐tasiRNA lines. [file TPJ-100-720-s003.pdf]

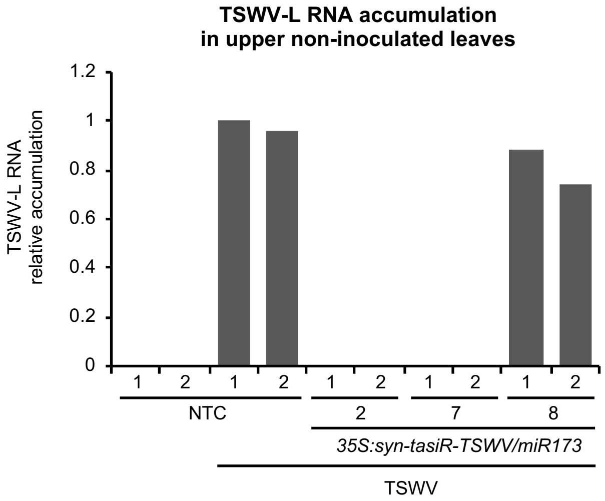

Figure S3. Relative TSWV-L RNA accumulation at 40 dpi in selected syn-tasiRNA lines and non-transgenic controls (NTCs) after normalization to *Solanym lycopersicum* actin (*Tom41*) and *Elongation Factor 1 alpha* (*eEF1a*), as determined by RT-qPCR (NTC infected plant 1 = 1.0).
