# Supplementary material for: Multi‐targeting of viral RNAs with synthetic trans‐acting small interfering RNAs enhances plant antiviral resistance
Source: Plant J. 2019 Sep 16;100(4):720–37. doi: 10.1111/tpj.14466 (PMC6899541; doi:10.1111/tpj.14466)
Supplement: Supplementary file 4 — Figure S4. Diagram of the complete 35S:amiR‐TSWV plasmid. [file TPJ-100-720-s004.pdf]

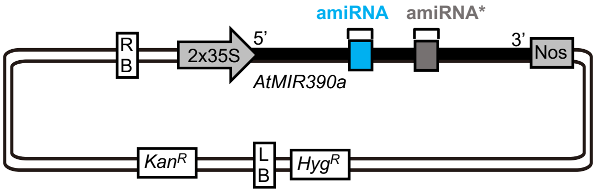

35S:amiR-TSWV

Figure S4. Diagram of the complete *35S:amiR-TSWV* plasmid. Other details are described in Figure 4a and Supporting Figure S1.
