# Supplementary material for: Multi‐targeting of viral RNAs with synthetic trans‐acting small interfering RNAs enhances plant antiviral resistance
Source: Plant J. 2019 Sep 16;100(4):720–37. doi: 10.1111/tpj.14466 (PMC6899541; doi:10.1111/tpj.14466)
Supplement: Supplementary file 6 — Figure S6 . Multiple alignment of all sequences corresponding to TSWV segment L found in databases. [file TPJ-100-720-s006.pdf]

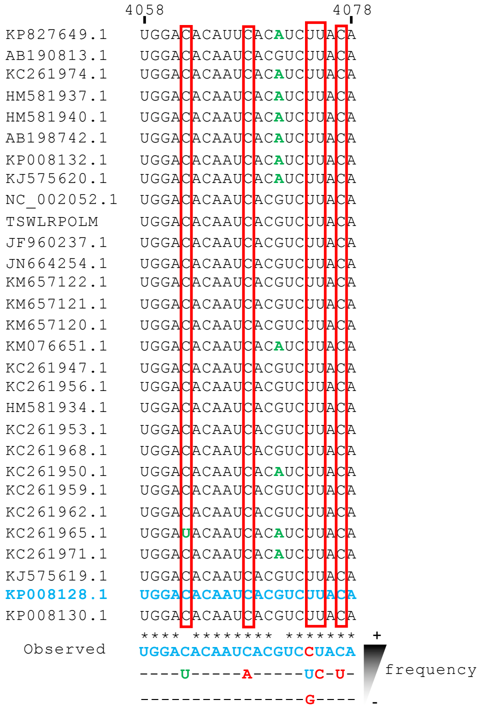

Figure S6. Multiple alignment of all sequences corresponding to TSWV segment L found in databases. Only the region comprising nucleotides 4058-4078 is shown. The name and sequence corresponding to the TSWV LL-N.05 isolate used in this study are in blue. Nucleotides found in other natural isolates or in TSWV progenies from infected amiRNA lines and differing from those of TSWV LL-N.05 are in green and red, respectively.
