# Supplementary material for: Multi‐targeting of viral RNAs with synthetic trans‐acting small interfering RNAs enhances plant antiviral resistance
Source: Plant J. 2019 Sep 16;100(4):720–37. doi: 10.1111/tpj.14466 (PMC6899541; doi:10.1111/tpj.14466)
Supplement: Supplementary file 7 — Table S1. Name, sequence and use of DNA oligonucleotides. [file TPJ-100-720-s007.docx]

**Table S1.** Name, sequence and use of DNA oligonucleotides.

| **Oligonucleotide** | **Sequence (5’->3’)** | **Use** |
| --- | --- | --- |
| AC-14 | atcgcgcgcggtgtcatctatgttactgaattcAAGCTTGGCGTGCCTGCA | Generation of *35S:syn-tasiR-TSWV/miR173* and *35S:syn-tasiR-GUS/miR173* constructs |
| AC-15 | ggaaacagctatgaccatgattacgaattcGAATTCAGTAACATAGATGACACCGCG |  |
| AC-49 | AGGACACAATCACGTCTTACA | amiR-TSWV/syn-tasiR-TSWV-1 detection |
| AC-50 | CCGATTCACCCAGAGCTGATA | syn-tasiR-TSWV-2 detection |
| AC-51 | CGGTATGCCCCACTATACCAA | syn-tasiR-TSWV-3 detection |
| AC-52 | AAGATGGATTGTGCACTCTGA | syn-tasiR-TSWV-4 detection |
| AC-55 | AGGGGCCATGCTAATCTTCTC | U6 detection |
| AC-203 | CTAAGCTAGATGCAATCTTCTCATA | RT-PCR and RT-qPCR amplification of TSWV-derived DNA including segment L target sites |
| AC-205 | GCACAAAGTGATCCATCGGAAG |  |
| AC-206 | GCAGAGATGAGCTTGAAAAGTCTG |  |
| AC-207 | GGCTCATTCTTTGATACTATCAGAGTG | RT-PCR amplification of TSWV-derived DNA including segment M target sites |
| AC-209 | CAGAGGGTTATGATCTTTCTGC |  |
| AC-210 | GCTTGCAAGCCTCAATGAGTG |  |
| AC-280 | CTAGGCTGGGTTCGCAGGAGATGATGC | qPCR amplification of tomato actin transcript |
| AC-281 | GTCTTTTTGACCCATACCCACCATCACAC |  |
| AC-282 | CCACCTCGAGATCCTAATGG | qPCR amplification of tomato Elongation Factor 1 alpha (*eEF1α*) transcript |
| AC-283 | ACCCTCACGTATGCTTCCAG |  |
| D-2698 | GTGATTTCTCTCTGCAAGCGAA | miR173 detection |
