# Supplementary material for: Multi‐targeting of viral RNAs with synthetic trans‐acting small interfering RNAs enhances plant antiviral resistance
Source: Plant J. 2019 Sep 16;100(4):720–37. doi: 10.1111/tpj.14466 (PMC6899541; doi:10.1111/tpj.14466)
Supplement: Supplementary file 8 — Table S2. Summary of results obtained from symptom and DAS‐ELISA analyses (upper non‐inoculated tissues) in Nicotiana benthamiana bioassays. [file TPJ-100-720-s008.docx]

**Table S2.** Summary of results obtained from symptom and DAS-ELISA analyses (upper non-inoculated tissues) in *Nicotiana benthamiana* bioassays.

|  | **Analysis at 10 dpi** | | **Analysis at 20 dpi** | |
| --- | --- | --- | --- | --- |
| **Sample** | **Symptomatic**  **plants/Total** | **DAS-ELISA**  **positive/Total** | **Symptomatic**  **plants/Total** | **DAS-ELISA**  **positive/Total** |
| *35S:GUS* | 0/6 | 0/6 | 0/6 | 0/6 |
| *35S:syn-tasiR-*  *G*US/miR173a + TSWV | 6/6 | 6/6 | 6/6 | 6/6 |
| *35S:syn-tasiR-*  *TSWV*/miR173a + TSWV | 0/6 | 0/6 | 1/6 | 1/6 |
| *35S:syn-tasiR-TSWV* +  *35S:MIR173a* + TSWV | 0/6 | 1/6 | 1/6 | 1/6 |
