# Supplementary material for: Multi‐targeting of viral RNAs with synthetic trans‐acting small interfering RNAs enhances plant antiviral resistance
Source: Plant J. 2019 Sep 16;100(4):720–37. doi: 10.1111/tpj.14466 (PMC6899541; doi:10.1111/tpj.14466)
Supplement: Supplementary file 9 — Appendix S1. DNA sequences in FASTA format of all artificial small RNA generating precursors used in this study. [file TPJ-100-720-s009.docx]

**Text S1.** DNA sequences in FASTA format of all artificial small RNA generating precursors used in this study.

(a) *AtMIR390a*-based amiRNA foldbacks. Sequence unique to the pri-*AtMIR390a*, pre-*AtMIR390a*, miRNA/amiRNA guide strand and miRNA*/amiRNA* strand sequences are highlighted in black, white, blue and green, respectively. Nucleotides of the pre-*AtMIR390a* that had to be modified to preserve the authentic *AtMIR390a* precursor structure are highlighted in red.

**>AtMIR390a**

TATAGGGGGGAAAAAAAGGTAGTCATCAGATATATATTTTGGTAAGAAAATATAGAAATGAATAATTTCACGTTTAACGAAGAGGAGATGACGTGTGTTCCTTCGAACCCGAGTTTTGTTCGTCTATAAATAGCACCTTCTCTTCTCCTTCTTCCTCACTTCCATCTTTTTAGCTTCACTATCTCTCTATAATCGGTTTTATCTTTCTCTAAGTCACAACCCAAAAAAACAAAGTAGAGAAGAATCTGTAAAGCTCAGGAGGGATAGCGCCATGATGATCACATTCGTTATCTATTTTTTGGCGCTATCCATCCTGAGTTTCATTGGCTCTTCTTACTACAATGAAAAAGGCCGAGGCAAAACGCCTAAAATCACTTGAGAATCAATTCTTTTTACTGTCCATTTAAGCTATCTTTTATAAACGTGTCTTATTTTCTATCTCTTTTGTTTAAACTAAGAAACTATAGTATTTTGTCTAAAACAAAACATGAAAGAACAGATTAGATCTCATCTTTAGTCTC

**>AtMIR390a-TSWV**

TATAGGGGGGAAAAAAAGGTAGTCATCAGATATATATTTTGGTAAGAAAATATAGAAATGAATAATTTCACGTTTAACGAAGAGGAGATGACGTGTGTTCCTTCGAACCCGAGTTTTGTTCGTCTATAAATAGCACCTTCTCTTCTCCTTCTTCCTCACTTCCATCTTTTTAGCTTCACTATCTCTCTATAATCGGTTTTATCTTTCTCTAAGTCACAACCCAAAAAAACAAAGTAGAGAAGAATCTGTATGTAAGACGTGATTGTGTCCTATGATGATCACATTCGTTATCTATTTTTTAGGACACAATAACGTCTTACACATTGGCTCTTCTTACTACAATGAAAAAGGCCGAGGCAAAACGCCTAAAATCACTTGAGAATCAATTCTTTTTACTGTCCATTTAAGCTATCTTTTATAAACGTGTCTTATTTTCTATCTCTTTTGTTTAAACTAAGAAACTATAGTATTTTGTCTAAAACAAAACATGAAAGAACAGATTAGATCTCATCTTTAGTCTC

(b) Sequence corresponding to miR173 target site is highlighted in blue. Sequence corresponding to tasiRNA-2 (position 3´D2[+]), tasiRNA-3 (position 3´D3[+]) and tasiRNA-4 (position 3´D4[+]) is highlighted in blue, green, dark and light pink, respectively. Sequence corresponding to the syn-tasiRNA cassette is highlighted in yellow. All the other sequences from Arabidopsis *TAS1c* gene are highlighted in black.

**>AtTAS1c**

AAACCTAAACCTAAACGGCTAAGCCCGACGTCAAATACCAAAAAGAGAAAAACAAGAGCGCCGTCAAGCTCTGCAAATACGATCTGTAAGTCCATCTTAACACAAAAGTGAGATGGGTTCTTAGATCATGTTCCGCCGTTAGATCGAGTCATGGTCTTGTCTCATAGAAAGGTACTTTCGTTTACTTCTTTTGAGTATCGAGTAGAGCGTCGTCTATAGTTAGTTTGAGATTGCGTTTGTCAGAAGTTAGGTTCAATGTCCCGGTCCAATTTTCACCAGCCATGTGTCAGTTTCGTTCCTTCCCGTCCTCTTCTTTGATTTCGTTGGGTTACGGATGTTTTCGAGATGAAACAGCATTGTTTTGTTGTGATTTTTCTCTACAAGCGAATAGACCATTTATCGGTGGATCTTAGAAAATTATTCTAAGTCCAACATAGCGTATTCTAAGTTCAACATATCGACGAACTAGAAAAGACATTGGACATATTCCAGGATATGCAAAAGAAAACAATGAATATTGTTTTGAATGTGTTCAAGTAAATGAGATTTTCAAGTCGTCTAAAGAACAGTTGCTAATACAGTTACTTATTTCAATAAATAATTGGTTCTAATAATACAAAACATATTCGAGGATATGCAGAAAAAAAGATGTTTGTTATTTTGAAAAGCTTGAGTAGTTTCTCTCCGAGGTGTAGCGAAGAAGCATCATCTACTTTGTAATGTAATTTTCTTTATGTTTTCACTTTGTAATTTTATTTGTGTTAATGTACCATGGCCGATATCGGTTTTATTGAAAGAAAATTTATGTTACTTCTGTTTTGGCTTTGCAATCAGTTATGCTAGTTTTCTTATACCCTTTCGTAAGCTTCCTAAGGAATCGTTCATTGATTTCCACTGCTTCATTGTATATTAAAACTTTACAACTGTATCGACCATCATATAATTCTGGGTCAAGAGATGAAAATAGAACACCACATCGTAAAGTGAAAT

**>AtTAS1c-TSWV**

AAACCTAAACCTAAACGGCTAAGCCCGACGTCAAATACCAAAAAGAGAAAAACAAGAGCGCCGTCAAGCTCTGCAAATACGATCTGTAAGTCCATCTTAACACAAAAGTGAGATGGGTTCTTAGATCATGTTCCGCCGTTAGATCGAGTCATGGTCTTGTCTCATAGAAAGGTACTTTCGTTTACTTCTTTTGAGTATCGAGTAGAGCGTCGTCTATAGTTAGTTTGAGATTGCGTTTGTCAGAAGTTAGGTTCAATGTCCCGGTCCAATTTTCACCAGCCATGTGTCAGTTTCGTTCCTTCCCGTCCTCTTCTTTGATTTCGTTGGGTTACGGATGTTTTCGAGATGAAACAGCATTGTTTTGTTGTGATTTTTCTCTACAAGCGAATAGACCATTTATCGGTGGATCTTAGAAAATTATGTAAGACGTGATTGTGTCCTTATCAGCTCTGGGTGAATCGGTTGGTATAGTGGGGCATACCGTCAGAGTGCACAATCCATCTTGAACTAGAAAAGACATTGGACATATTCCAGGATATGCAAAAGAAAACAATGAATATTGTTTTGAATGTGTTCAAGTAAATGAGATTTTCAAGTCGTCTAAAGAACAGTTGCTAATACAGTTACTTATTTCAATAAATAATTGGTTCTAATAATACAAAACATATTCGAGGATATGCAGAAAAAAAGATGTTTGTTATTTTGAAAAGCTTGAGTAGTTTCTCTCCGAGGTGTAGCGAAGAAGCATCATCTACTTTGTAATGTAATTTTCTTTATGTTTTCACTTTGTAATTTTATTTGTGTTAATGTACCATGGCCGATATCGGTTTTATTGAAAGAAAATTTATGTTACTTCTGTTTTGGCTTTGCAATCAGTTATGCTAGTTTTCTTATACCCTTTCGTAAGCTTCCTAAGGAATCGTTCATTGATTTCCACTGCTTCATTGTATATTAAAACTTTACAACTGTATCGACCATCATATAATTCTGGGTCAAGAGATGAAAATAGAACACCACATCGTAAAGTGAAAT

**>AtTAS1c-GUS**

AAACCTAAACCTAAACGGCTAAGCCCGACGTCAAATACCAAAAAGAGAAAAACAAGAGCGCCGTCAAGCTCTGCAAATACGATCTGTAAGTCCATCTTAACACAAAAGTGAGATGGGTTCTTAGATCATGTTCCGCCGTTAGATCGAGTCATGGTCTTGTCTCATAGAAAGGTACTTTCGTTTACTTCTTTTGAGTATCGAGTAGAGCGTCGTCTATAGTTAGTTTGAGATTGCGTTTGTCAGAAGTTAGGTTCAATGTCCCGGTCCAATTTTCACCAGCCATGTGTCAGTTTCGTTCCTTCCCGTCCTCTTCTTTGATTTCGTTGGGTTACGGATGTTTTCGAGATGAAACAGCATTGTTTTGTTGTGATTTTTCTCTACAAGCGAATAGACCATTTATCGGTGGATCTTAGAAAATTATATTGACCCACACTTTGCCGATAACCTTCACCCGGTTGCCACTATTGACCCACACTTTGCCGATAACCTTCACCCGGTTGCCACGAACTAGAAAAGACATTGGACATATTCCAGGATATGCAAAAGAAAACAATGAATATTGTTTTGAATGTGTTCAAGTAAATGAGATTTTCAAGTCGTCTAAAGAACAGTTGCTAATACAGTTACTTATTTCAATAAATAATTGGTTCTAATAATACAAAACATATTCGAGGATATGCAGAAAAAAAGATGTTTGTTATTTTGAAAAGCTTGAGTAGTTTCTCTCCGAGGTGTAGCGAAGAAGCATCATCTACTTTGTAATGTAATTTTCTTTATGTTTTCACTTTGTAATTTTATTTGTGTTAATGTACCATGGCCGATATCGGTTTTATTGAAAGAAAATTTATGTTACTTCTGTTTTGGCTTTGCAATCAGTTATGCTAGTTTTCTTATACCCTTTCGTAAGCTTCCTAAGGAATCGTTCATTGATTTCCACTGCTTCATTGTATATTAAAACTTTACAACTGTATCGACCATCATATAATTCTGGGTCAAGAGATGAAAATAGAACACCACATCGTAAAGTGAAAT
