# Supplementary material for: Multi‐targeting of viral RNAs with synthetic trans‐acting small interfering RNAs enhances plant antiviral resistance
Source: Plant J. 2019 Sep 16;100(4):720–37. doi: 10.1111/tpj.14466 (PMC6899541; doi:10.1111/tpj.14466)
Supplement: Supplementary file 10 [file TPJ-100-720-s010.docx]

**Figure S1.** Diagram of the complete *35S:syn-tasiR-TSWV/miR173* plasmid. Approximate locations of T-DNA right and left borders (RB and LB, respectively) as well as bacterial and plant antibiotic resistance genes (kanamycin and hygromycin, respectively) are indicated. Other details are described in Figure 1a.

**Figure S2.** Diagram of the *35S:syn-tasiR-GUS/miR173* construct. *TAS1c* precursor from *A. thaliana* (in black) was engineered to produce four anti-GUS syn-tasiRNAs (in light or dark greyboxes). Base pairing between syn-tasiRNA and TSWV nucleotides (in grey and black, respectively) is shown. Specific cleavage sites in target sites located in GUS RNAs are indicated with black arrows, with TS coordinates indicated in brackets. Other details are described in Figure 1a.

**Figure S3.** Relative TSWV-L RNA accumulation at 40 dpi in selected syn-tasiRNA lines and non-transgenic controls (NTCs) after normalization to *Solanym lycopersicum* actin (*Tom41*) and *Elongation Factor 1 alpha* (*eEF1α*), as determined by RT-qPCR (NTC infected plant 1 = 1.0).

**Figure S4.** Diagram of the complete *35S:amiR-TSWV* plasmid. Other details are described in Figure 4a and Supplemental Figure S1.

**Figure S5.** Relative TSWV-L RNA accumulation at 40 dpi in selected amiRNA lines and non-transgenic controls (NTCs) after normalization to *Solanym lycopersicum* actin (*Tom41*) and *Elongation Factor 1 alpha* (*eEF1α*), as determined by RT-qPCR (NTC infected plant 1 = 1.0).

**Figure S6.** Multiple alignment of all sequences corresponding to TSWV segment L found in databases. Only the region comprising nucleotides 4058-4078 is shown. The name and sequence corresponding to the TSWV LL-N.05 isolate used in this study are in blue. Nucleotides found in other natural isolates or in TSWV progenies from infected amiRNA lines and differing from those of TSWV LL-N.05 are in green and red, respectively.

**Table S1.** Name, sequence and use of DNA oligonucleotides.

**Table S2.** Summary of results obtained from symptom and DAS-ELISA analyses (upper non-inoculated tissues) in *Nicotiana benthamiana* bioassays.

**Text S1.** DNA sequences in FASTA format of all artificial small RNA generating precursors used in this study.

(a) *AtMIR390a*-based amiRNA foldbacks. Sequence unique to the pri-*AtMIR390a*, pre-*AtMIR390a*, miRNA/amiRNA guide strand and miRNA*/amiRNA* strand sequences are highlighted in black, white, blue and green, respectively. Nucleotides of the pre-*AtMIR390a* that had to be modified to preserve the authentic *AtMIR390a* precursor structure are highlighted in red.

(b) Sequence corresponding to miR173 target site is highlighted in blue. Sequence corresponding to tasiRNA-2 (position 3´D2[+]), tasiRNA-3 (position 3´D3[+]) and tasiRNA-4 (position 3´D4[+]) is highlighted in blue, green, dark and light pink, respectively. Sequence corresponding to the syn-tasiRNA cassette is highlighted in yellow. All the other sequences from Arabidopsis *TAS1c* gene are highlighted in black.
